# An integrative methodology based on protein-protein interaction networks for identification and functional annotation of disease-relevant genes applied to channelopathies

**DOI:** 10.1186/s12859-019-3162-1

**Published:** 2019-11-12

**Authors:** Milagros Marín, Francisco J. Esteban, Hilario Ramírez-Rodrigo, Eduardo Ros, María José Sáez-Lara

**Affiliations:** 1 Department of Computer Architecture and Technology – CITIC, University of Granada, Granada, Spain; 2 Systems Biology Unit, Department of Experimental Biology, University of Jaén, Jaén, Spain; 3 Department of Biochemistry and Molecular Biology I, University of Granada, Granada, Spain

**Keywords:** Channelopathies, Protein-protein interaction networks, Genotype-phenotype relationships, Translational bioinformatics, Behavioural diagnosis, Genetic diseases, Systems medicine

## Abstract

**Background:**

Biologically data-driven networks have become powerful analytical tools that handle massive, heterogeneous datasets generated from biomedical fields. Protein-protein interaction networks can identify the most relevant structures directly tied to biological functions. Functional enrichments can then be performed based on these structural aspects of gene relationships for the study of channelopathies. Channelopathies refer to a complex group of disorders resulting from dysfunctional ion channels with distinct polygenic manifestations. This study presents a semi-automatic workflow using protein-protein interaction networks that can identify the most relevant genes and their biological processes and pathways in channelopathies to better understand their etiopathogenesis. In addition, the clinical manifestations that are strongly associated with these genes are also identified as the most characteristic in this complex group of diseases.

**Results:**

In particular, a set of nine representative disease-related genes was detected, these being the most significant genes in relation to their roles in channelopathies. In this way we attested the implication of some voltage-gated sodium (SCN1A, SCN2A, SCN4A, SCN4B, SCN5A, SCN9A) and potassium (KCNQ2, KCNH2) channels in cardiovascular diseases, epilepsies, febrile seizures, headache disorders, neuromuscular, neurodegenerative diseases or neurobehavioral manifestations. We also revealed the role of Ankyrin-G (ANK3) in the neurodegenerative and neurobehavioral disorders as well as the implication of these genes in other systems, such as the immunological or endocrine systems.

**Conclusions:**

This research provides a systems biology approach to extract information from interaction networks of gene expression. We show how large-scale computational integration of heterogeneous datasets, PPI network analyses, functional databases and published literature may support the detection and assessment of possible potential therapeutic targets in the disease. Applying our workflow makes it feasible to spot the most relevant genes and unknown relationships in channelopathies and shows its potential as a first-step approach to identify both genes and functional interactions in clinical-knowledge scenarios of target diseases.

**Methods:**

An initial gene pool is previously defined by searching general databases under a specific semantic framework. From the resulting interaction network, a subset of genes are identified as the most relevant through the workflow that includes centrality measures and other filtering and enrichment databases.

## Background

The genetic aetiology of many complex diseases comprises different specific clinical symptoms and evolution. The identification of their causal agents becomes essential for the detection of suitable targets, the management of their diagnosis and the selection of the most adequate therapies [1–3]. The increasing availability of large bibliographic data volumes lays the foundations for the identification of these candidate genes [2, 4]. However, the integration of all this knowledge requires understanding the diverse biomedical information sources available. The extraction of data performed by valid association procedures and the comprehensive interpretation of all this current knowledge is complex. This is in and of itself an issue of utmost importance for the purpose mentioned above [4–6].

Traditional reductionist strategies that deal with this diverse wealth of information focus on the study of particular molecules or signalling pathways that are useful for the identification of diagnostic biomarkers. Nevertheless, it does not seem enough to approach all the system complexity [2, 4]. Alternatively, interdisciplinary research is developing new technologies and integrative computational methodologies in order to better understand pathogeneses [7, 8]. Some studies that use these current integrative methodologies allow the discovery of co-morbidities between Alzheimer’s disease and some types of cancers [9] where genetic factors can play an important role along with other factors such as the environment, lifestyle, and drug treatments. They are also being used to perform a genome-wide search for Autism gene candidates [1]. These new tools are able to manage deductive analyses by gaining insight into the connections among diseases, even between those *a priori* not related by the traditional bibliographic searches, which usually tend to be subjective, time-consuming or not reproducible [10]. However, the large range of diverse new tools created within different focuses hinders the existence of a unique approach to or a consensus on their usage. Thus, data extraction through ad-hoc approaches using specific tools may again be complex, not reproducible or subjective. In this way, network analyses and functional annotation tools represent some of the best strategies for objective interpretation of biomedical data and cope with higher level of biological complexity [1–3, 11].

The identification of relevant genes is being addressed from the global analysis of multiple interactions at different levels, usually employing networks as representations of the biological complex interactions underlying clinical disorders [11–13]. A way to systematically decode the cellular signalling networks consists in the identification of interactome for the detection of the central nodes which maintain the structure and information fluxes into the functional network [11, 14]. Despite some limitations, protein-protein interaction (PPI) networks have been suitably applied to the definition of biological mechanisms by integrating PPI data with transcriptional changes [1, 13–15]. It is evidenced that in disease networks in which the alteration is produced by mutations, the node or nodes mutated play a primary role in the development of diseases and thus have a central position in the network [16]. In the case of multifactorial diseases, the nodes which seem to be the causal factor could be located in the periphery. However, the key nodes in the main biological and molecular processes affected, i.e. potential pharmacological targets, tend to have a central position in the network [1, 17–19]. Thus, for the identification of the most significant genes in a disease as molecular targets there are useful software tools of high impact [20–24]. One of them is STRING [22], a database used to build predicted and well-known PPI networks. The interactions in STRING are mainly derived from automated text-mining and databases of previous knowledge, among other resources. Other well-known tool is Cytoscape [23], an open-source software platform which has being designed for the purpose of visualizing, analysing and modelling complex biological networks and pathways.

Furthermore, in a system biology approach it is highly important to know the biological and molecular processes in which the complex set of genes involved play a joint key role. Though, if the aim were to identify pharmacological targets, it would also be mandatory to unveil if these candidate genes could also be related with other diseases as comorbidity [9, 25]. These annotations and associations can be performed through traditional bibliographic search systems, which are inefficient, subjective and time consuming by hand [10], or by using some of the highest impact tools from the large number of platforms developed for functional annotation in objective, quick and reproducible ways [26]. This is the case of DAVID [24], which has been shown to provide an automatic comprehensive set of functional annotation tools for biological interpretation of large gene lists as pharmacological targets [7]. It is also very useful in unveiling other related diseases, providing a more comprehensive view of the importance of treatments [9, 19].

In this regard, the aim of this study is to present a semi-automatic workflow using PPI networks for the identification and functional annotation of the most relevant genes in diseases. This new contribution to the extant methods is based on the integration of a set of multidimensional data from different biological levels (genomics, transcriptomics and proteomics) in order to analyse genetic correlations among diseases with different clinical symptomatologies and/or clinical prognoses (and still based on similar molecular mechanisms). In order to illustrate the value of this integrative approach and demonstrate its usefulness, we applied this methodology to the case of channelopathies as proof-of-concept in order to understand their most common polygenic influences, which contributes to the overall understanding of pathomechanisms underlying these altered-channels diseases, in how mutations can modify disease severity [27] and to shed some light on effective treatments [19, 28, 29]. We showed that this proposed workflow is able to mine current available databases and platforms in the context of channelopathies.

## Results

**Fig. 1:**
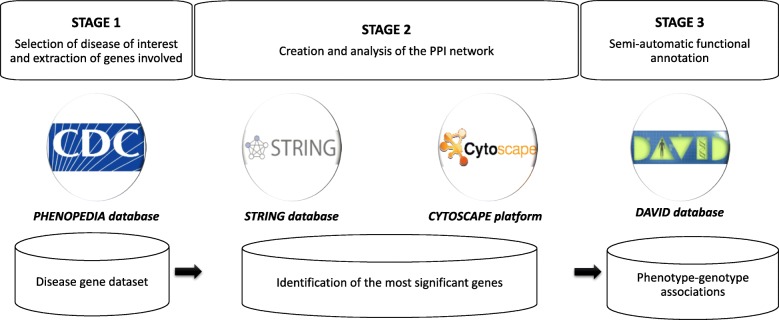
Semi-automatic workflow for the identification and functional annotation of the most relevant genes in a pathology. Stage 1) *Phenopedia* [30] is a disease-centered view of genetic association studies summarized in the online *Human Genome Epidemiology (HuGE) encyclopedia*. It provides a list of genes involved in the disease of interest. It is in this stage that a complex disease, a set of diseases or a certain disease can be chosen to be studied. Stage 2) *STRING* [22] is a database of known and predicted protein-protein interactions that allows the discovery of relationships across disease genotype and thus the creation of the PPI network. *Cytoscape* [23] is an open source software platform for the visualization and analysis of complex networks that measures each gene and identifies network nodes. Stage 3) *DAVID* database [24] works as a semi-automatic functional annotation tool of the genes obtained after Stage 2

In this section, we illustrate the experimental application of the semi-automatic workflow (Fig. 1) to the case of channelopathies.

### Semi-automatic workflow applied to channelopathies

#### Gene dataset of the disease under study

First, the gene dataset of channelopathies was created by introducing the term “channelopathies” in the first stage of the present workflow (Fig. 1 Stage 1), which generated a list of 42 genes involved in this complex group of disorders: *SCN5A, KCNH2, KCNQ1, HLA-B, RYR2, SCN2A, SCN4A, CACNA1C, KCNE1, KCNE2, CACNA1S, ATP8B4, DCHS1, SCN4B, SCN2B, SCN9A, SNTA1, CDKL5, STK11, STXBP1, TGFB1, TGFB2, TRPC4, SCN1A, SCN1B, HLA-DRB5, HSPB2, KCNQ2, LOXL2, CNGB3, SCN3B, PCDH19, KCNE3, AKAP9, PRRT2, CLCN1, ASB10, ARX, DMPK, SPESP1, ANK3, HLA-A.*

#### Identification of the most relevant genes

**Fig. 2:**
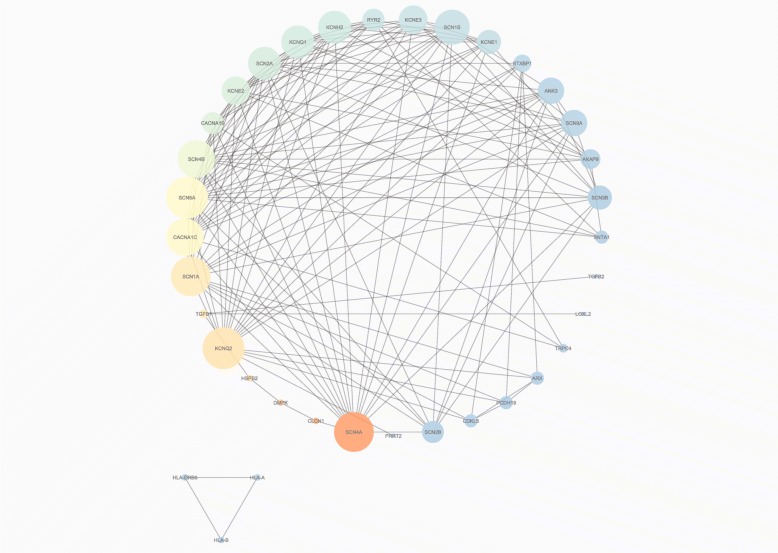
Protein-protein interaction (PPI) network of channelopathies analysed in Stage 2. Each network node represents the protein produced by each single, protein-coding gene locus from the gene dataset of channelopathies. The representation is a circular layout based on the betweenness attribute with undirected edges (other layouts shown in Additional file 10). The node size marks the level of degree and therefore neighbourhood (the larger nodes represent proteins with a higher number of interactions). The node colour shows the level of betweenness and therefore the level of centrality (the warmer the colour of the protein, the shorter path between the two which indicates how important the node is within the wider context of the entire network) [31]. HLA proteins are discarded due to their disconnection from the principal component. The image was generated by Cytoscape [23]

**Fig. 3:**
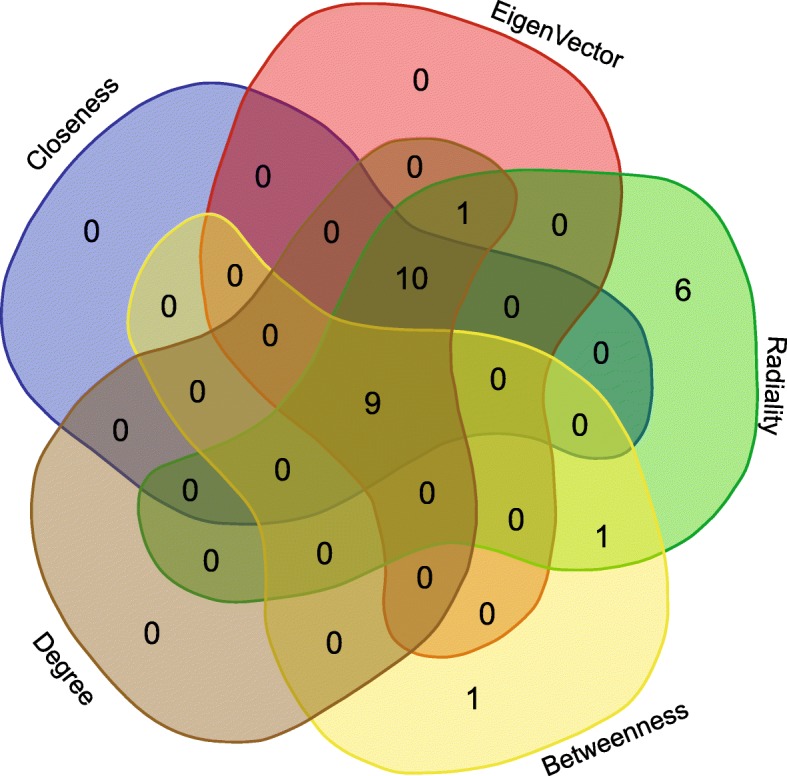
Nine genes as the most relevant in Stage 2 using different centrality statistics. Venn diagram representing the intersections calculated through the use of other statistics for the proposed centrality measures such as closeness, Eigenvector and radiality. The same set of genes identified with degree and betweenness only still turned out to be the most relevant in channelopathies: SCN1A, SCN2A, SCN4A, SCN4B, SCN5A, SCN9A, KCNH2, KCNQ2 and ANK3. Venn diagram was obtained using a free available tool provided by Ghent University [32]. All the statistics information is included in Additional file 2

Then, the list of gene names was the input for Stage 2 (Fig. 1. Stage 2). Our target organism was *H. sapiens*, and a PPI network was generated through the STRING database (interactome network presented in Additional file 1) and then analysed by the Cytoscape platform (Fig. 2). We employed the main features used as centrality parameters, degree and betweenness (as described in methods) for the identification of the most important vertices within the graph. Thus, starting from 42 genes involved in channelopathies, nine genes with the highest degree of interactions and betweenness in their gene expressions were stemmed as the most relevant in channelopathies: SCN9A, ANK3, SCN5A, SCN2A, KCNQ2, SCN1A, KCNH2, SCN4B and SCN4A. This same set of nine relevant genes was also obtained using other connectivity features, such as closeness, EigenVector and radiality (Fig. 3). The result proves to be robust and concordant with that from Stage 2 of the workflow using only betweenness and centrality.

#### Gene functional annotation

Finally, the functional annotation of each gene was automatically generated in Stage 3 using DAVID search tool (Fig. 1. Stage 3). All the functional annotation results are detailed in section 4.1 in Additional file 4.

### Validation of the workflow

To measure the quality of the results obtained, we carried out an alternative more conventional search with a view to comparing the workflow annotation results to the results offered by two other widespread family of bibliographic methods, such as systematic review and exhaustive review.

#### Comparison criterion

Using “MeSH” ontology [33], we selected four upper-level categories with their corresponding lower-level ones. Each of these lower-level categories refers to one or more diseases linked to these genes. We used “health disorder” as the specific comparator which contains up to four upper-level categories: 1) cardiovascular diseases, 2) nervous system diseases, 3) mental diseases, and 4) other diseases. This frame comprises all the phenotypes of each relevant gene in channelopathies to facilitate the visualization and comparison of functional annotation results (as specified in Table 1). In Additional file 3 we can find the “MeSH”-based terminological hierarchies of the selection of the lower-level categories.

**Table 1:** “MeSH”-based categories selected. A total of four upper-level categories and their corresponding lower-level categories capture all the phenotypes manifested by more than one of these genes. We resorted to “MeSH” terminological-based hierarchical networks that include all the phenotypes as referred in the third column (included in Additional file 3)

Upper-level category	Lower-level category	Hierarchical network
Cardiovascular diseases	Vascular diseases	Figure 3.1 in Additional file 3
	Cardiac arrhythmias	
	Other diseases (heart arrest, cardiomyopathies, myocardial ischemia or cardiomegaly)	
Nervous system diseases	Neurobehavioral manifestations	Figure 3.2 in Additional file 3
	Febrile seizures	
	Epilepsy	
	Headache disorders	
	Neurodegenerative diseases	
	Neuromuscular diseases	
Mental disorders	Tobacco use disorder	Figure 3.3 in Additional file 3
	Other mental disorders (bipolar disorder, Alzheimer disease, autism, depression or schizophrenia)	
Other disorders	Sudden death	Figure 3.4 in Additional file 3
	Diabetes Mellitus type 2	
	Periodic paralyses	

From the functional annotation results through the last stage of the proposed workflow (using DAVID) (Table 4.1.8 in Additional file 4) and applying “health disorder” as the specific-domain category, we obtained the results (consigned in Table 4.2.1 in Additional file 4) that will be visually represented in the final results of this work (Figs. 5, 6 and 7).

#### Systematic review and exhaustive review as other traditional search systems

**Fig. 4:**
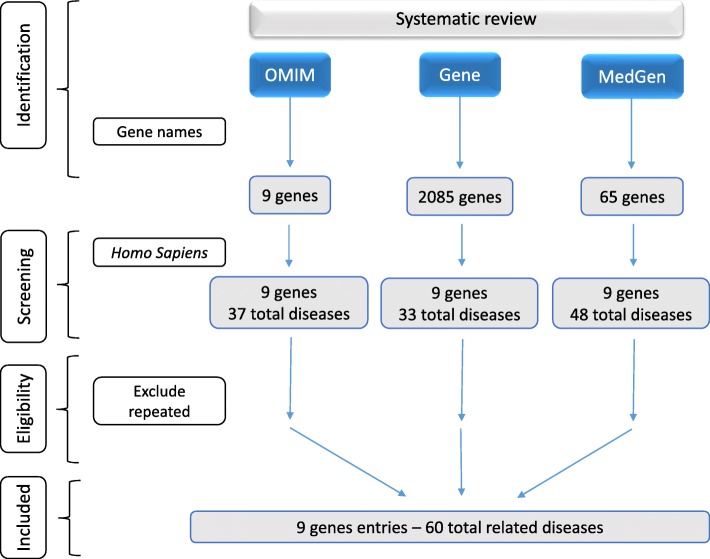
Systematic review procedure for the genes of interest through OMIM, Gene and MedGen databases

In the systematic review we searched by phenotype nomenclatures, filtered by *H. sapiens* as the target organism and removed duplicate entries (Fig. 4). Finally, we extracted nine gene entries from the OMIM and Gene databases and 32 evidences of diseases from the MedGene database (Table 2; all the diseases extracted through the systematic review can be found in Additional file 5). Following the same four upper-level categories, we created an equivalent table containing each disease or clinical manifestation related with its corresponding genes. We used “health disorder” as the specific-domain category and obtained the results shown in Additional file 6. We also compared DAVID against other phenotype-oriented databases of high impact, proving again the selection of this tool in Stage 3 (information included in Additional file 9).

**Table 2:** Gene accession numbers filtered through systematic review

GENE	OMIM ID	Gene ID
SCN1A	182389	6323
SCN2A	182390	6326
SCN4A	603967	6329
SCN4B	608256	6330
SCN5A	600163	6331
SCN9A	603415	6335
KCNQ2	602235	3785
KCNH2	152427	3757
ANK3	600465	288

As our third step, exhaustive review was performed by using the query words “gene product nomenclature” + “diseases” in the search box of PubMed and MEDLINE resources, the evidence filtering being the most time-consuming task. We took the same categories and created an equivalent table containing each disease or clinical manifestation related to the corresponding genes, its “health disorder” as the specific-domain category, and its bibliographic references (Additional file 7). While performing this traditional review, we could also expand the functional annotation of the most relevant genes with further information, detailed in Additional file 8.

#### Representation through genotype-phenotype association networks

**Fig. 5:**
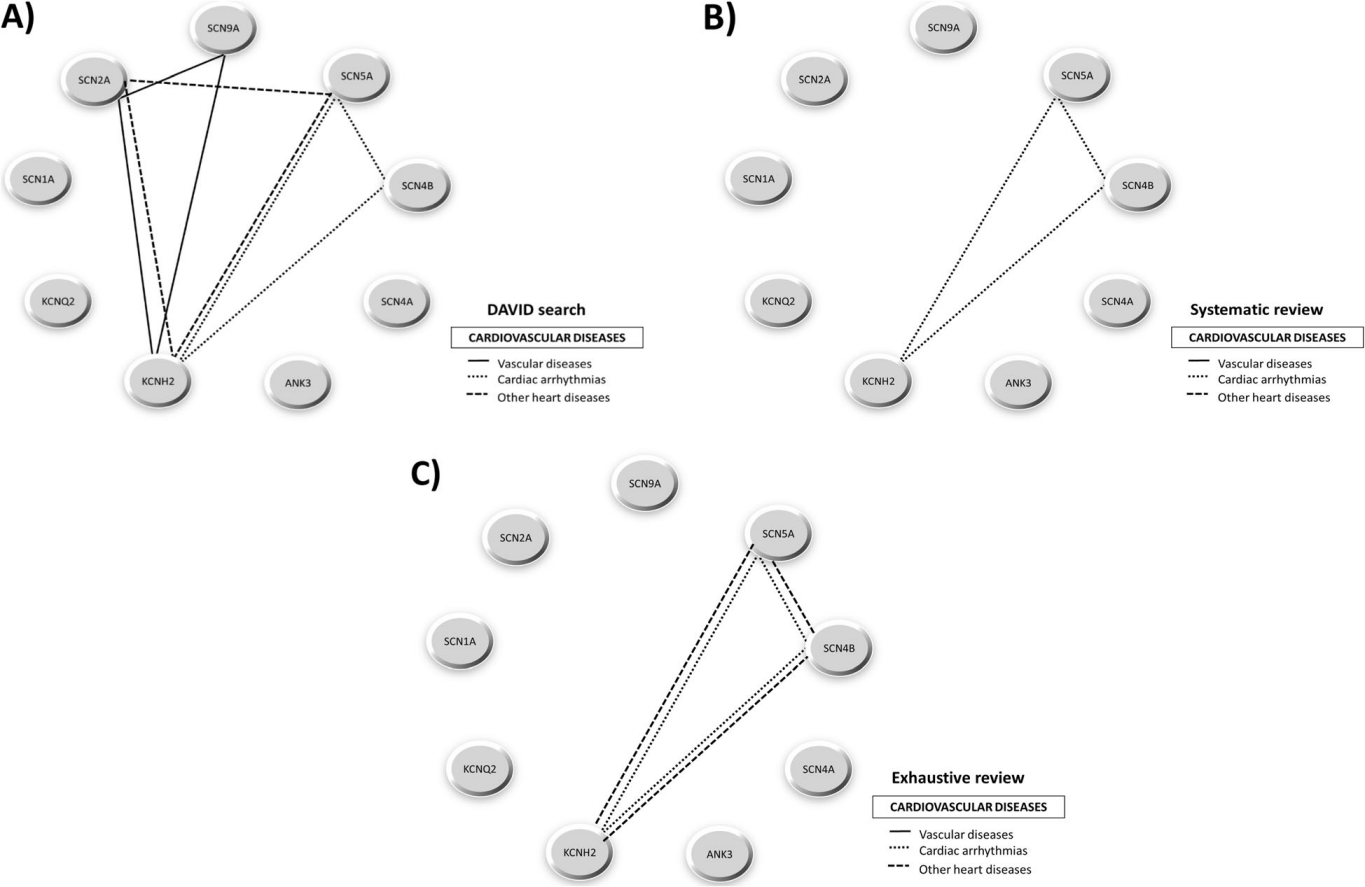
Genotype-phenotype association network for cardiovascular diseases category**.** Association networks created for cardiovascular diseases category from the evidences obtained by **a**) last stage of the workflow, **b**) systematic review and **c**) exhaustive review. The “MeSH”-based categories include each pathophysiological evidence shared by two or more genes. Other heart diseases include heart arrest, cardiomyopathies, myocardial ischemia or cardiomegaly

**Fig. 6:**
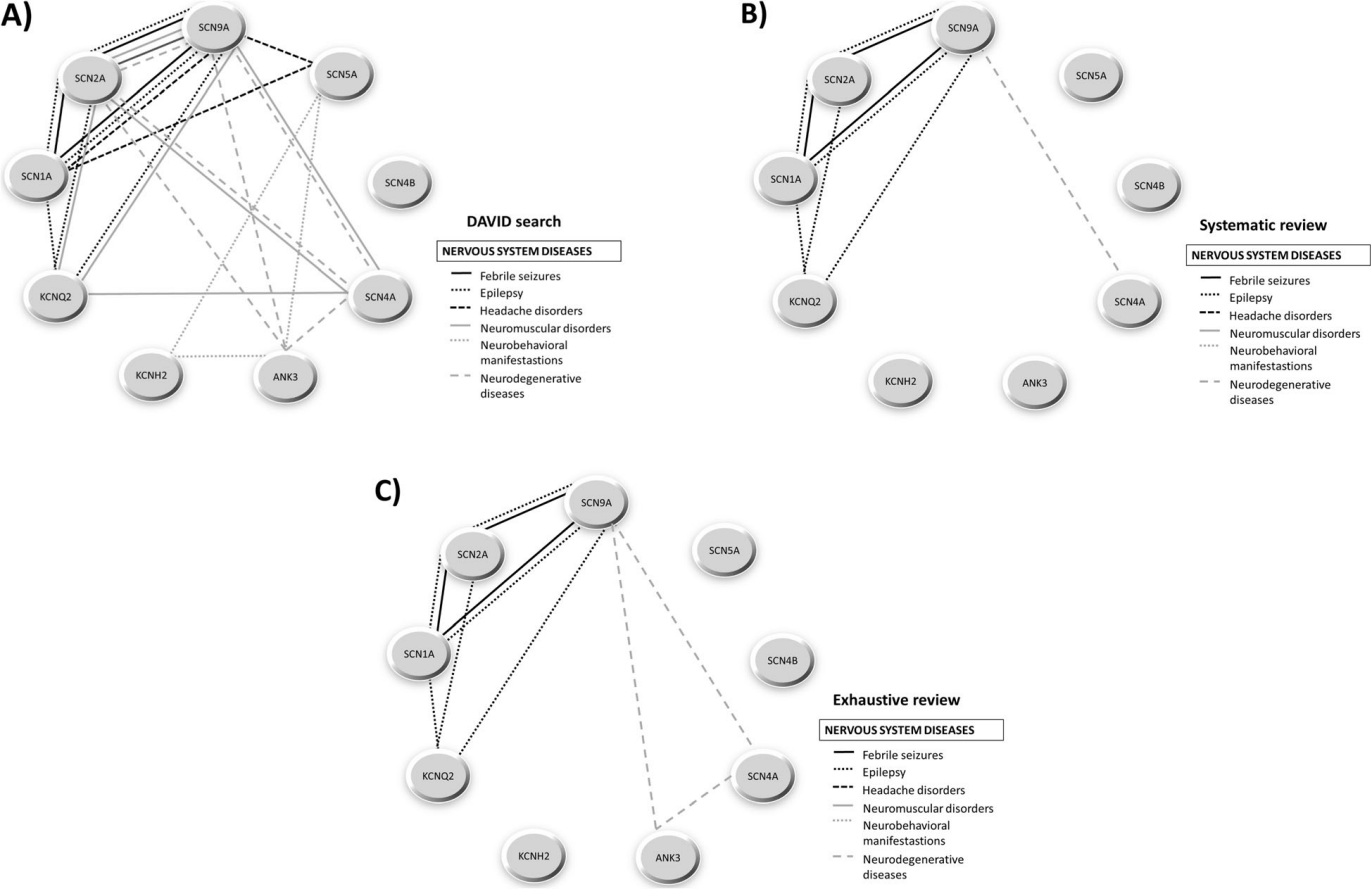
Genotype-phenotype association network for nervous system diseases category**.** Association networks created for nervous system diseases category from the evidences obtained by **a**) last stage of the workflow, **b**) systematic review and **c**) exhaustive review. The “MeSH”-based categories comprise each pathophysiological evidence shared by two or more genes

**Fig. 7:**
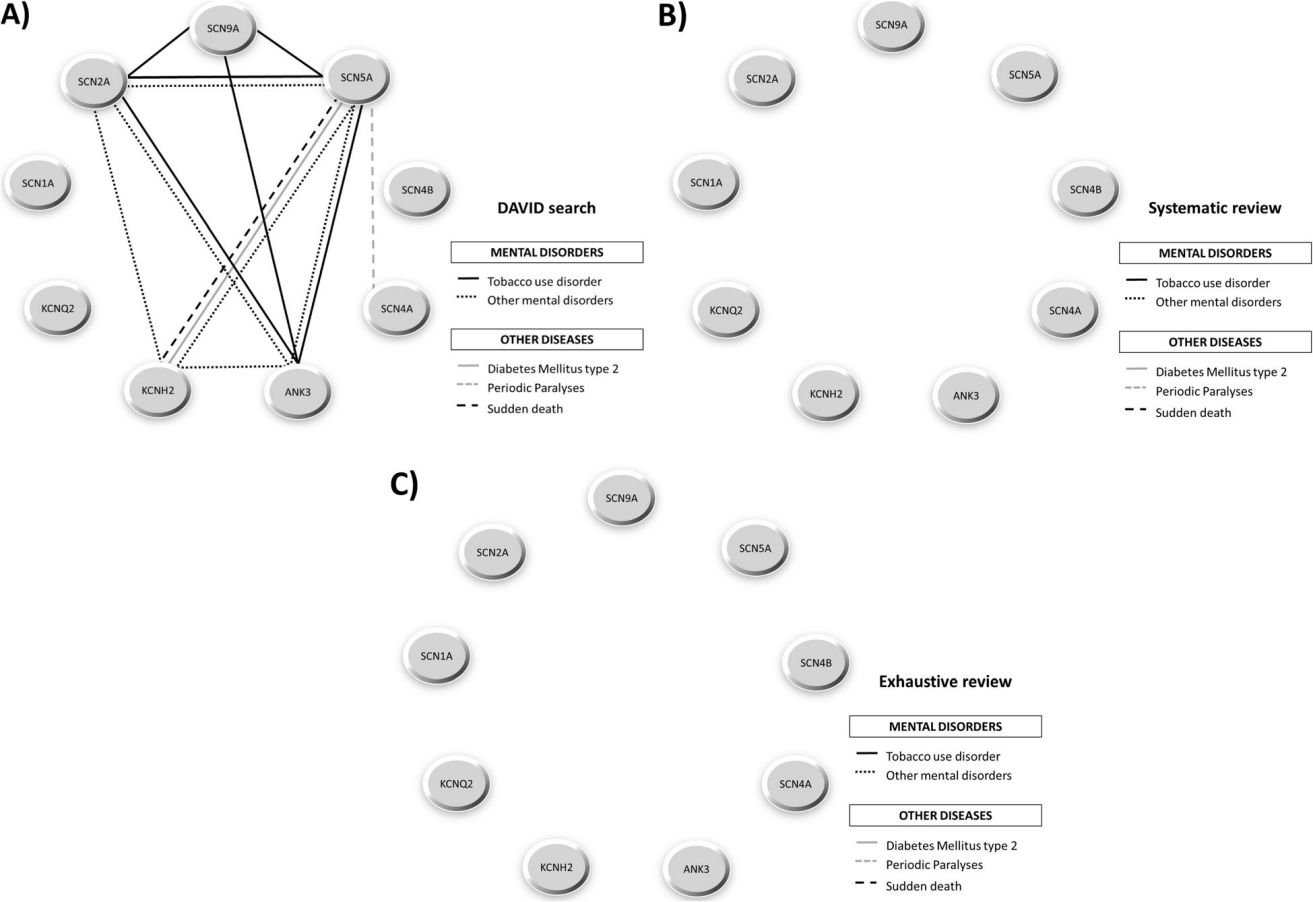
Genotype-phenotype association network for mental and other diseases categories**.** Association networks created for mental and other diseases categories from the evidences obtained by **a**) last stage of the workflow, **b**) systematic review and **c**) exhaustive review. The “MeSH”-based categories comprise each pathophysiological evidence shared by two or more genes. Other mental disorders include bipolar disorder, Alzheimer disease, autism, depression or schizophrenia

From the genotype-phenotype relationships found by the three search systems used in this work – the last stage of the workflow (Table 4.2.1 in Additional file 4), the systematic review (Additional file 5), and the exhaustive review (Additional file 7) — and considering all the categories selected for every phenotype, we represented association networks for cardiovascular diseases (Fig. 5), nervous system diseases (Fig. 6), and mental diseases and other disorders (Fig. 7).

For cardiovascular diseases, DAVID search (Fig. 5a) found more diseases than the systematic review (Fig. 5b) and the exhaustive review (Fig. 5c), with the exception of a connection between the gene SCN4B and “other heart diseases” category retrieved by the exhaustive review but not by DAVID or systematic searches. This is due to the fact that the gene product of SCN4B is an auxiliary subunit, hence it influences but not directly causes the disease. In fact, it has been found to be associated with various inherited arrhythmia syndromes (Brugada syndrome, long-QT syndrome type 3, progressive cardiac conduction defect, sick sinus node syndrome, atrial fibrillation, and dilated cardiomyopathy) [34]. For nervous system diseases, DAVID search (Fig. 6a) provided many more phenotypic connections among genes than systematic review (Fig. 6b) or exhaustive review (Fig. 6c), which obtained the same amount of information. In fact, we could observe that the only gene with a lack of disease association is the SCN4B which, as mentioned above, is associated with cardiovascular diseases only. Finally, for mental and other disorders we only found phenotypic connections for the most relevant genes in DAVID (Fig. 7a), but not through systematic review (Fig. 7b) nor exhaustive review (Fig. 7c).

## Discussion

In the present study, we addressed the prediction of the most relevant genes in the context of a group of pathologies not necessarily homogeneous but linked by a common term, as is the case of channelopathies. The identification of those genes may present several shortcomings: 1) finding key genes through scientific literature might be a burdensome task due to the fuzzy and textual nature of information, 2) completely objective criteria are hard to define, and 3) the comparison and validation of different search methodologies might not be objectively carried out. To tackle limitation 1), we developed an integrative methodology using a workflow which departs from genes linked to particular diseases. Then we built a protein-protein interaction network from which key genes are identified through the determination of the centrality measures. Finally, we proceeded to functionally annotate these key genes through the application of widely used data analysis tools in the bibliography.

Although the proposed methodology is of general purpose, in this study it was applied to the set of diseases termed channelopathies. In this clinical context, our method allowed the identification of the most relevant genes (with the highest degree of intermediation and centrality) related to channelopathies. The products of these genes are mostly channels of two different types, namely voltage-gated sodium channels — SCN1A, SCN2A, SCN4A, SCN4B, SCN5A, and SCN9A — that are involved in the rapid depolarisation in the cardiac conduction (Reactome ID: R-HSA-5576892, Table 4.1.6 in Additional file 4), and voltage-gated potassium channels — KCNQ2 and KCNH2 — responsible for the activation of the voltage-gated potassium channels family in the neuronal system (Reactome ID: R-HSA-1296072, Table 4.1.6. in Additional file 4) [35–37]. KCNH2 is also involved in the rapid repolarisation of the cardiac conduction (Reactome ID: R-HSA-5576890, Table 4.1.6. in Additional file 4). On the other hand, Ankyrin-G (ANK3) is a protein which deals with the vesicle-mediated transport of the membrane trafficking (Reactome ID: R-HSA-374562, Table 4.1.6. in Additional file 4) and is also responsible for linking integral membrane proteins such as the voltage-gated sodium channel with the spectrin-based membrane skeleton [38]. Particularly, all the genes except KCNH2 contribute to the interaction between cytoskeleton adaptor ankyrins and a type of adhesion receptor (L1) which inhibits the nerve growth at the neural development pathway (Reactome ID: R-HSA-445095, Table 4.1.6. in Additional file 4) [35, 39].

Defects in the ion channels throughout the human body have been involved in a wide phenotypic variability in channelopathies. This remarkable causal heterogeneity makes the diseases hard to classify [40]. Some reviews deal with the categorization of channelopathies based on the organ system with which they are mainly associated in both clinical and pathophysiological aspects [28, 40–43]. Other reviews opt to classify channelopathies according to the ion channel proteins in order to improve the understanding of how their specific mutations can be linked to diseases [27, 44–46]. In current reviews the implication of voltage-gated sodium channels with cardiac pathologies (such as long-QT syndrome and fatal arrhythmias) and epilepsies is easily retrievable [27]. The role of some voltage-gated potassium channels with cardiac pathologies (heart arrhythmias, dilated cardiomyopathies), epilepsies and chronic pain is also well studied [27]. On the contrary, we do not know much about the clustering of Ankyrin-G at the axonal initial segments in the nervous system with voltage-gated sodium channels [47, 48] and some potassium channels [49]. In our work we found this implication of voltage-gated sodium and potassium channels in cardiovascular diseases (SCN2A-SCN9A-KCNH2 cluster for vascular diseases, SCN2A-SCN5A-KCNH2 cluster for cardiac arrhythmias and SCN5A-SCN4B-KCNH2 cluster for other heart diseases) (Fig. 5). We also discovered a very high interconnection and participation of the genes selected not only in epilepsies, but also in febrile seizures, headache disorders, neuromuscular and neurodegenerative diseases and neurobehavioral manifestations (Fig. 6). It is interesting to highlight that in our results the above mentioned participation of Ankyrin-G in the nervous system (Fig. 6) is also reflected, specifically in neurobehavioral manifestations (ANK3-SCN5A-KCNH2 cluster) and neurodegenerative diseases (ANK3-SCN2A-SCN4A-SCN9A cluster). Finally, our results showed the implication of the genes obtained in other types of diseases, such as tobacco use disorder, diabetes mellitus type 2 or sudden death (Fig. 7), which consequently means the involvement of these genes in other systems, such as the immunological system [50] or the endocrine system [40]. As discussed above, we found that these results corroborate the conclusions collected by current literature about channelopathies, even outcomes which are not retrievable in comparative terms with respect to other traditional literature mining.

Approaching the above-mentioned validation of the proposed methodology by statistical comparison with other extant methods would be difficult due to their very different nature and properties. For that reason, we compared our proposal with two traditional and widespread family of methods, these being systematic review and exhaustive review. Among the three methods employed, our workflow and the systematic review proved to be the most objective approach when compared to the exhaustive review. Our results indicate that our methodology is actually able to find more correlations among the nine genes selected than any of the other two methods. Particularly, the present approach allows the detection of many more correlations than the systematic review (as seen in Figs. 5, 6 and 7).

Therefore, the proposed methodology is able to gather as much significant information as any other traditional literature search system mentioned in this work. At the same time, it was shown to work more flexibly, making it a convenient and easy-to-perform first-level approach compared to the above-mentioned methods.

## Conclusion

We showed the usefulness of a semi-automatic integrative workflow with regard to successful, currently available mining databases and platforms based on protein-protein interaction networks applied to channelopathies. This workflow builds as productive results as a non-automatic research but in a quicker way, functioning as a bridge-builder among fields and allowing the extraction of information which *a priori* might not seem relevant when the starting point is a very large group of genes in disease. We encourage future line of research to focus on the full automatization of the workflow and the use of more specific statistical resources such as principal component analysis or machine learning classifiers.

## Methods

In this section, we present the semi-automatic workflow (Fig. 1) and describe the current systems biology tools and processes used. Thus, the course of action runs as follows: first, a gene dataset of disease under study is extracted; second, a protein-protein interaction network is built and analysed and the most significant genes in disease are selected; third, the functional annotation for each relevant gene is performed.

### Semi-automatic workflow

#### Gene dataset of the disease under study

In the first step of the workflow (Fig. 1, Stage 1), the “MeSH” term [33] of the disease at issue was obtained to know the unequivocal medical concept and introduced in Phenopedia [30]. Phenopedia is an online tool provided by the Center for Disease Control and Prevention (CDC) which allows linking genomic discoveries with health care and disease prevention. Through Phenopedia we extracted the list of genes which have been demonstrated to be involved in the disease so far.

#### Identification of the most relevant genes

The next step (Fig. 1, Stage 2) consisted in the generation of a protein-protein interaction (PPI) network from the list of genes through STRING [22]. We considered *Homo sapiens* as the target organism and extracted the PPI network. Then, the NetworkAnalizer available in Cytoscape [23] allows to compute and analyse a comprehensive set of topological parameters. The most highly connected proteins with a central role in the network are three times more likely to be essential than those with peripheral role, while at the same time being more associated with alterations that have a primary role in the development of diseases [51]. The identification of relevant genes in a disease has been addressed using two centrality parameters for the detection of the central nodes which maintain the structure and information fluxes into the functional network [17, 52, 53]. The network centrality features considered in the proposed workflow are degree and betweenness, two fundamental parameters in graph theory [17, 51–53]. Centrality degree is defined as the number of interactions in which a protein is involved. Betweenness is the number of shortest paths between all pairs of other proteins that pass through a certain protein [52, 53]. We set a threshold on both centrality parameters by their means and, after sorting them, those gene expressions exceeding this threshold were selected as the most relevant genes (Additional file 2).

#### Gene functional annotation

The last stage employed DAVID search (Fig. 1 Stage 3) for the functional annotation of genes, allowing the description of their main biological processes and the development of a functional enrichment analysis (providing information about Gene Ontology, protein interactions, functional protein domains, diseases associations, and signalling pathways, among others) from a list of genes (as official gene symbols) for the target organism *Homo sapiens.*

### Validation of the workflow

Genotype-phenotype relationships of genes were obtained through the classification of the pathophysiological manifestations and diseases associated to the genes at issue. For the validation of the present workflow, we mapped those genotype-phenotype relationships of the genes obtained from the functional annotation onto phenotypic networks. We considered specific-domain category “health disorder” as our choice of interest from all the functional annotation results. This category was taken from the Medical Subject Headings (“MeSH”), a terminological database that captures biomedical information through ontological hierarchies [33]. MeSH offers a hierarchical organization of different pathological categories of every clinical manifestation that facilitates the representation of genotype-phenotype relationships. The pathophysiological implications shared by the most significant genes can thus be easily identified by means of their grade of intermediation and interaction.

Hence, we could compare the results of this workflow with the clinical manifestations associated to these genes through the use of two current traditional bibliographic search systems, systematic and exhaustive reviews (and other phenotype-oriented resources, Additional file 9). We followed the guidelines of the International Union of Pharmacology [54, 55] for the gene products nomenclature. Then we created genotype-phenotype association networks for each disease to clearly illustrate their relationships, helping visualize at a glance the different phenotypes found for every gene and thus to be able to validate the efficiency in the extraction of significant information by the presented methodology. Those diseases with no more than one gene associated were purposefully omitted in the network.

DAVID bases its disease annotation search on two human gene databases: Online Mendelian Inheritance in Man (OMIM, URL: /www.omim.org/) and Genetic Association Database from complex diseases and disorders (GAD DISEASE, URL: /geneticassociationdb.nih.gov/). The systematic review was performed using databases that focus on the relationships found between human genotypes and phenotypes of genetic alterations. Web resources for data presented herein are Online Mendelian Inheritance in Man (OMIM, URL: www.omim.org/); Gene, which integrates information about phenotypes and associated conditions (URL: www.ncbi.nlm.nih.gov/gene/); and MedGene, which offers search results about human medical genetics and conditions related to the genetic contribution (URL: www.ncbi.nlm.nih.gov/medgen/) (Fig. 4). The exhaustive review is sometimes an evidence-based review, more extensive and also takes much more time and significant effort than the systematic review, making it a tedious process in terms of filtering and selection of information. It is usually carried out by using a search equation with key words defining an unspecific question of interest [10].

## Supplementary information

**Additional file 1.** Protein-protein interaction (PPI) network obtained from the list of gene names involved in channelopathies. Each network node represents the protein produced by each single, protein-coding gene locus (Image generated by STRING). All the nodes are coloured to show that they are the query proteins used as input for the STRING platform. The nodes which are filled represent that some 3D structure is known or predicted; empty nodes do not present any 3D structure discovered as yet. The edges indicate protein-protein associations (full legend available in STRING).

**Additional file 2.** Connectivity statistics calculated from the PPI network. Raw statistics values of the two main centrality measures (degree and betweenness) are considered in Stage 2 of the workflow. Other connectivity features (closeness, Eigenvector and radiality) are included as evidence of the efficiency of the workflow and robustness of the results. The same nine genes identified as the most relevant are obtained from the average calculation of all these features. This intersection was represented in Fig. 3. HLA proteins were discarded due to their disconnection from the principal component, as shown in Additional file 1 and Fig. 2.

**Additional file 3.** Description of the upper-level and lower-level categories selected for the creation of “MeSH”-based terminological networks. Hierarchical trees of the upper-level categories (cardiovascular diseases, nervous system diseases, mental diseases, and other diseases) are described in detail. Lower-level categories are based on disease evidences obtained through DAVID search, systematic review and exhaustive review. The categories selected for the genotype-phenotype representations are highlighted in grey.

**Additional file 4.** Functional annotation results obtained through Stage 3 of the workflow. Section 1 refers to all the raw functional annotation results of the most relevant genes in channelopathies directly extracted from the last stage of the workflow (DAVID search). Section 2 refers to the extraction of the diseases from this source of information. The diseases then were classified by their lower-level categories according to the “MeSH” criterion described in methods. Some evidences could not be classified due to lack of enough information. These categories will allow the visual representation of genotype-phenotype associations obtained through DAVID search.

**Additional file 5.** Procedure and data extracted through the systematic review of the most relevant genes in channelopathies. Detailed procedure for the filtering and extraction of the relevant information of each gene and its diseases involved in channelopathies after the application of the systematic review.

**Additional file 6.** Dataset of genotype-phenotype relationships found through systematic review of the most relevant genes in channelopathies. Diseases related to the nine relevant genes through the systematic review based on three databases (Gene, OMIM and MedGen). Each phenotype is classified according to its MeSH category, as described in methods.

**Additional file 7.** Dataset of genotype-phenotype relationships found through exhaustive review of the most relevant genes in channelopathies. Diseases linked to the nine relevant genes through the exhaustive review. Each phenotype is classified according to its MeSH category, as described in methods. Some evidences cannot be classified due to lack of information.

**Additional file 8.** Further functional annotation results after the exhaustive review of the most relevant genes in channelopathies. Summary of the localization, distribution and functions of the genes after the exhaustive review, as well as a summary of further information found in this review. CNS: Central Nervous System; PNS: Peripheral Nervous System; DRG: dorsal root ganglion.

**Additional file 9 **Quantitative validation by significance analysis of DAVID search against other phenotype-oriented resources. We searched the nine relevant genes resulted from the workflow in PheGenI [56], ToppGene [57] and g:Profiler [58]. We quantitatively evaluated this search selecting those terms with a significance less than 0.05 using Benjamini-Hochberg FDR statistic. We obtained a minor result in DAVID search (OMIM search did not offer the phenotypes *p*-values, unlike GAP DISEASE database). Even so, results are useful to develop a quantitative comparison between semiautomatic platforms and bibliographic search systems (sheet 1). From these results we represented the genotype-phenotype association networks to compare easily each p-value phenotype obtained (sheet 2). It should be noted that p-values of clinical phenotypes could be only obtained from one of the two databases explored through DAVID (GAP DISEASE database), and so the genotype-phenotype association network is sparser than the network of the manuscript (section A in Figs. 5, 6, 7). Yet, it is demonstrated that the workflow results are statistically significant and are as valid as or even better than systematic or exhaustive reviews. Then, we created three Boolean tables (in sheets 3, 4, 5) comparing each phenotype obtained from each search; these tables were then converted to binary matrices and clustering multivariate statistical analyses and bootstrap validations were carried out. This approach demonstrated that the results provided in the manuscript, obtained from DAVID (DAVID_m) and systematic and exhaustive reviews, clustered together in a robust and significant way (sheets 3, 4, 5). Hence, this workflow builds as productive results as a non-automatic research but in a quicker way allowing the extraction of information which *a priori* might not seem relevant when the starting point is a very large group of genes in disease. Moreover, the results obtained using just significant FDR corrected *p*-values also cluster in particular branches.

**Additional file 10.** Protein-protein interaction (PPI) network of channelopathies analysed in Stage 2 represented by other layouts. The PPI network of channelopathies is represented as a circular layout with the betweenness attribute (Fig. 2), or with the degree attribute in the first page of this file. We also included this network with a hierarchical layout in the second page of this file as other type of representation of the same dense network. Both types of representations present each node with undirected edges. The node size marks the level of degree and therefore of neighbourhood (the larger nodes represent proteins with a higher number of interactions). The node colour shows the level of betweenness and therefore the level of centrality. HLA proteins are discarded due to their disconnection from the principal component. The images were generated by Cytoscape [23].

## Data Availability

All data analysed during this study are included in this published article [and its supplementary information files].

## Abbreviations

BioGRID: Biological General Repository for Interaction Datasets
DAVID: Database for Annotation, Visualization and Integrated Discovery
HuGE: Human Genome Epidemiology
MeSH: Medical Subject Headings
OMIM: Online Mendelian Inheritance in Man
PPI: Protein-protein interaction
