# Supplementary figures and images for: An integrative methodology based on protein-protein interaction networks for identification and functional annotation of disease-relevant genes applied to channelopathies
Source: BMC Bioinformatics. 2019 Nov 12;20:565. doi: 10.1186/s12859-019-3162-1 (PMC6849233; doi:10.1186/s12859-019-3162-1)

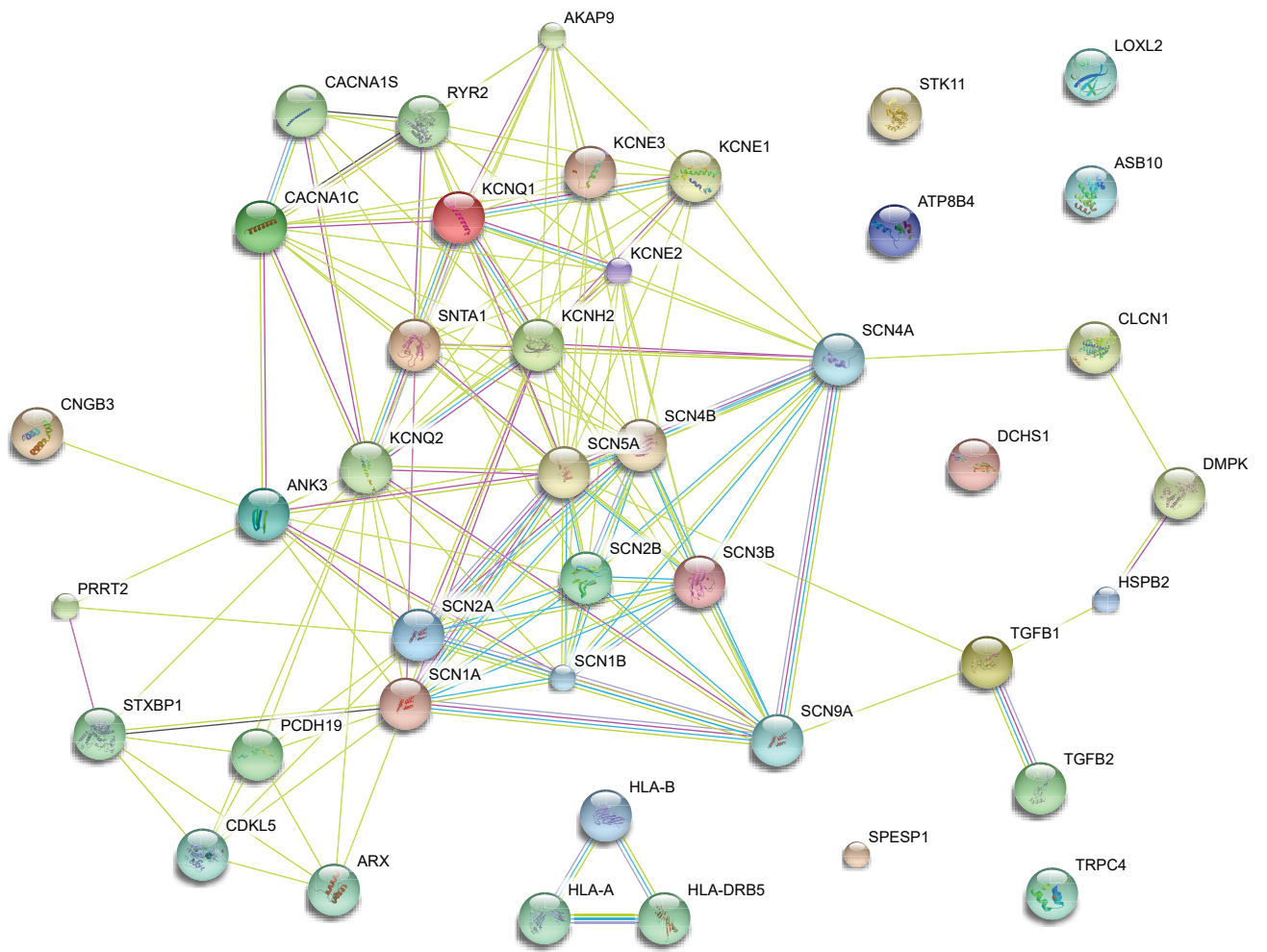

Supplement: Supplementary file 1 — Additional file 1. Protein-protein interaction (PPI) network obtained from the list of gene names involved in channelopathies. Each network node represents the protein produced by each single, protein-coding gene locus (Image generated by STRING). All the nodes are coloured to show that they are the query proteins used as input for the STRING platform. The nodes which are filled represent that some 3D structure is known or predicted; empty nodes do not present any 3D structure discovered as yet. The edges indicate protein-protein associations (full legend available in STRING). [file 12859_2019_3162_MOESM1_ESM.pdf]

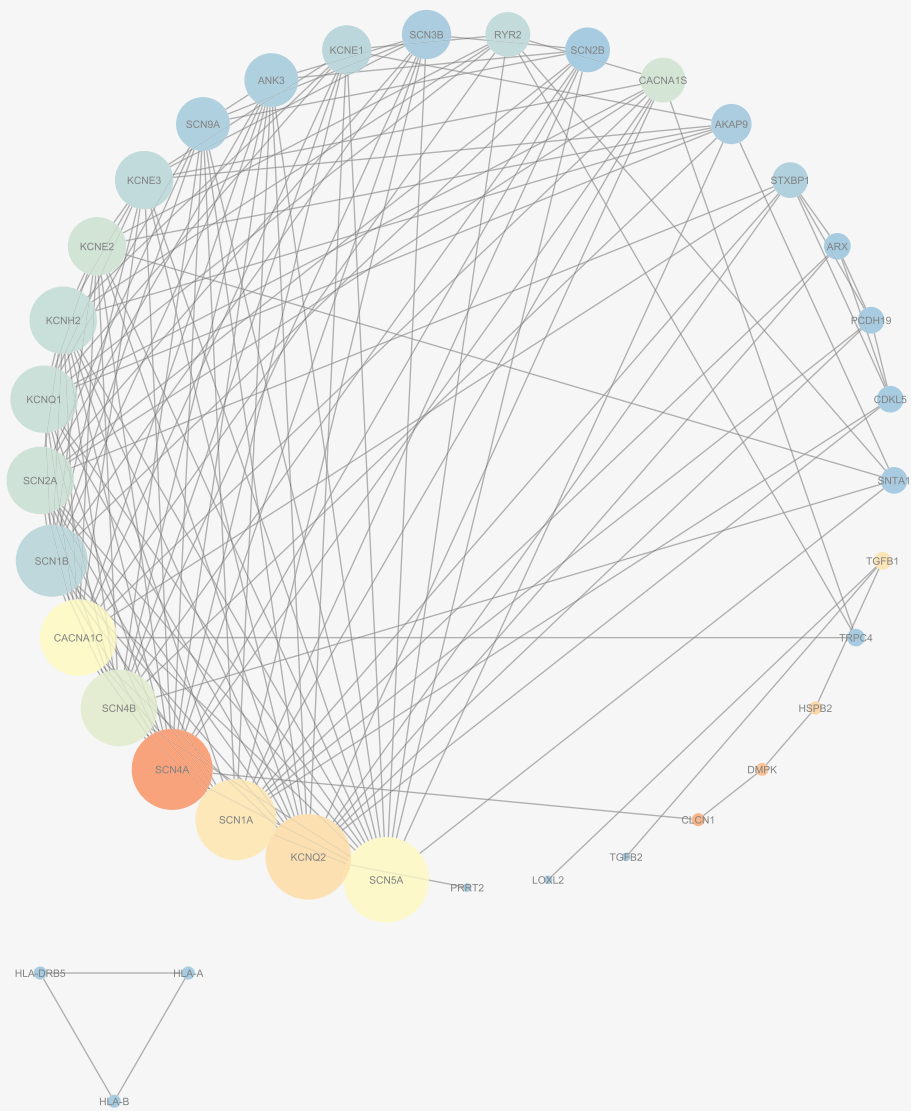

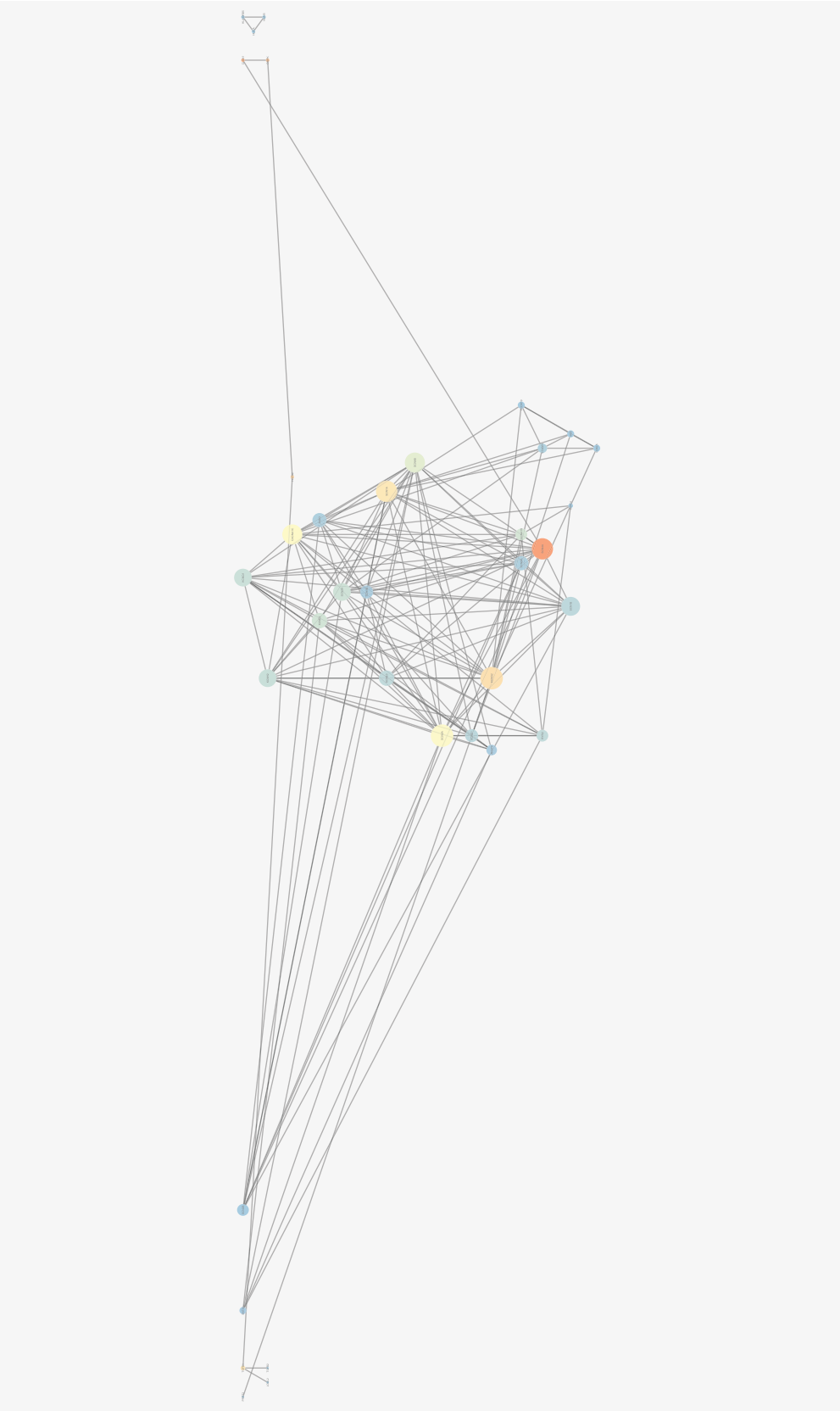

Supplement: Supplementary file 10 — Additional file 10. Protein-protein interaction (PPI) network of channelopathies analysed in Stage 2 represented by other layouts. The PPI network of channelopathies is represented as a circular layout with the betweenness attribute (Fig. 2), or with the degree attribute in the first page of this file. We also included this network with a hierarchical layout in the second page of this file as other type of representation of the same dense network. Both types of representations present each node with undirected edges. The node size marks the level of degree and therefore of neighbourhood (the larger nodes represent proteins with a higher number of interactions). The node colour shows the level of betweenness and therefore the level of centrality. HLA proteins are discarded due to their disconnection from the principal component. The images were generated by Cytoscape [23]. [file 12859_2019_3162_MOESM10_ESM.pdf]
