# Supplementary material for: An integrative methodology based on protein-protein interaction networks for identification and functional annotation of disease-relevant genes applied to channelopathies
Source: BMC Bioinformatics. 2019 Nov 12;20:565. doi: 10.1186/s12859-019-3162-1 (PMC6849233; doi:10.1186/s12859-019-3162-1)
Supplement: Supplementary file 2 — Additional file 2. Connectivity statistics calculated from the PPI network. Raw statistics values of the two main centrality measures (degree and betweenness) are considered in Stage 2 of the workflow. Other connectivity features (closeness, Eigenvector and radiality) are included as evidence of the efficiency of the workflow and robustness of the results. The same nine genes identified as the most relevant are obtained from the average calculation of all these features. This intersection was represented in Fig. 3. HLA proteins were discarded due to their disconnection from the principal component, as shown in Additional file 1 and Fig. 2. [file 12859_2019_3162_MOESM2_ESM.pdf]

name	Betweenness unDir	name	Degree unDir	name	Closeness unDir	name	EigenVector unDir	name	Radiality unDir
**KCNQ2**	0.172	**KCNQ2**	19	HLA-B	0.500	**KCNH2**	0.304	**SCN5A**	4.147
**SCN5A**	0.158	**SCN5A**	18	HLA-DRB5	0.500	**SCN5A**	0.295	**KCNH2**	4.059
**TGFB1**	0.129	**KCNH2**	18	HLA-A	0.500	**SCN4B**	0.274	**KCNQ2**	4.059
**SCN4A**	0.115	**SCN4B**	16	**SCN5A**	0.022	**KCNQ2**	0.254	**SCN4B**	4.029
**SCN1A**	0.080	**SCN1A**	14	**KCNH2**	0.021	**KCNE2**	0.241	**KCNE2**	3.912
**ANK3**	0.067	**CACNA1C**	13	**KCNQ2**	0.021	**KCNQ1**	0.225	**SCN2B**	3.912
**SCN9A**	0.059	**KCNE2**	13	**SCN4B**	0.020	**CACNA1C**	0.222	**SCN1A**	3.912
**CLCN1**	0.052	**SCN4A**	13	**KCNE2**	0.019	**SCN4A**	0.221	**KCNQ1**	3.882
**SCN2A**	0.049	**KCNE3**	12	**SCN2B**	0.019	**SCN3B**	0.217	**SCN3B**	3.882
**KCNH2**	0.044	**KCNQ1**	12	**SCN1A**	0.019	**KCNE3**	0.216	**KCNE3**	3.882
**SCN4B**	0.032	**SCN2A**	12	**KCNQ1**	0.019	**AKAP9**	0.208	**SCN1B**	3.882
SCN2B	0.022	**SCN2B**	12	**SCN3B**	0.019	**KCNE1**	0.207	**SCN9A**	3.882
CACNA1C	0.020	**SCN3B**	12	**KCNE3**	0.019	**SNTA1**	0.205	**KCNE1**	3.853
KCNE3	0.015	**AKAP9**	11	**SCN1B**	0.019	**SCN2B**	0.203	**SCN2A**	3.853
SCN1B	0.015	**KCNE1**	11	**SCN9A**	0.019	**SCN1A**	0.199	**SNTA1**	3.824
KCNE2	0.013	**SCN1B**	11	**KCNE1**	0.018	**SCN1B**	0.190	**CACNA1C**	3.794
HSPB2	0.013	**SNTA1**	11	**SCN2A**	0.018	**SCN2A**	0.177	**AKAP9**	3.794
SCN3B	0.011	**SCN9A**	10	**SNTA1**	0.018	**SCN9A**	0.166	**SCN4A**	3.794
SNTA1	0.009	**ANK3**	9	**CACNA1C**	0.018	**RYR2**	0.161	**ANK3**	3.765
KCNQ1	0.009	**RYR2**	9	**AKAP9**	0.018	**ANK3**	0.146	**RYR2**	3.588
KCNE1	0.008	CACNA1S	6	**SCN4A**	0.018	CACNA1S	0.109	**CACNA1S**	3.559
RYR2	0.004	CDKL5	6	**ANK3**	0.017	CDKL5	0.064	**TGFB1**	3.500
DMPK	0.004	PCDH19	6	RYR2	0.016	PCDH19	0.064	**CDKL5**	3.441
STXBP1	0.004	STXBP1	6	CACNA1S	0.015	STXBP1	0.054	**PCDH19**	3.441
AKAP9	0.004	ARX	5	TGFB1	0.015	ARX	0.051	**STXBP1**	3.441
CACNA1S	0.003	TGFB1	4	CDKL5	0.014	PRRT2	0.039	**ARX**	3.412
CDKL5	0.001	PRRT2	3	PCDH19	0.014	TGFB1	0.037	**PRRT2**	3.324
PCDH19	0.001	CLCN1	2	STXBP1	0.014	CLCN1	0.018	CLCN1	3.029
PRRT2	0.001	DMPK	2	ARX	0.014	CNGB3	0.012	CNGB3	2.882
ARX	0.000	HLA-A	2	PRRT2	0.014	HSPB2	0.003	HSPB2	2.735
CNGB3	0.000	HLA-B	2	CLCN1	0.012	TGFB2	0.003	TGFB2	2.618
HLA-A	0.000	HLA-DRB5	2	CNGB3	0.011	DMPK	0.002	DMPK	2.324
HLA-B	0.000	HSPB2	2	HSPB2	0.011	HLA-A	0.000	HLA-B	0.294
HLA-DRB5	0.000	CNGB3	1	TGFB2	0.010	HLA-DRB5	0.000	HLA-DRB5	0.294
TGFB2	0.000	TGFB2	1	DMPK	0.009	HLA-B	0.000	HLA-A	0.294
Mean	0.032	Mean	8.743	Mean	0.017	Mean	0.122	Mean	3.251
