# Supplementary material for: An integrative methodology based on protein-protein interaction networks for identification and functional annotation of disease-relevant genes applied to channelopathies
Source: BMC Bioinformatics. 2019 Nov 12;20:565. doi: 10.1186/s12859-019-3162-1 (PMC6849233; doi:10.1186/s12859-019-3162-1)

**Figure 3.1 Representation of the MeSH categories comprising all the cardiovascular diseases associated to the genes.** Main categories shared among genes are selected and highlighted in grey: 1) vascular diseases; 2) cardiac arrhythmias; 3) Other heart diseases which include: heart arrest, cardiomyopathies, myocardial ischemia or cardiomegaly. These lower-level categories comprise the “MeSH” terminological-based network of genotype-phenotype associations about cardiovascular diseases.

^
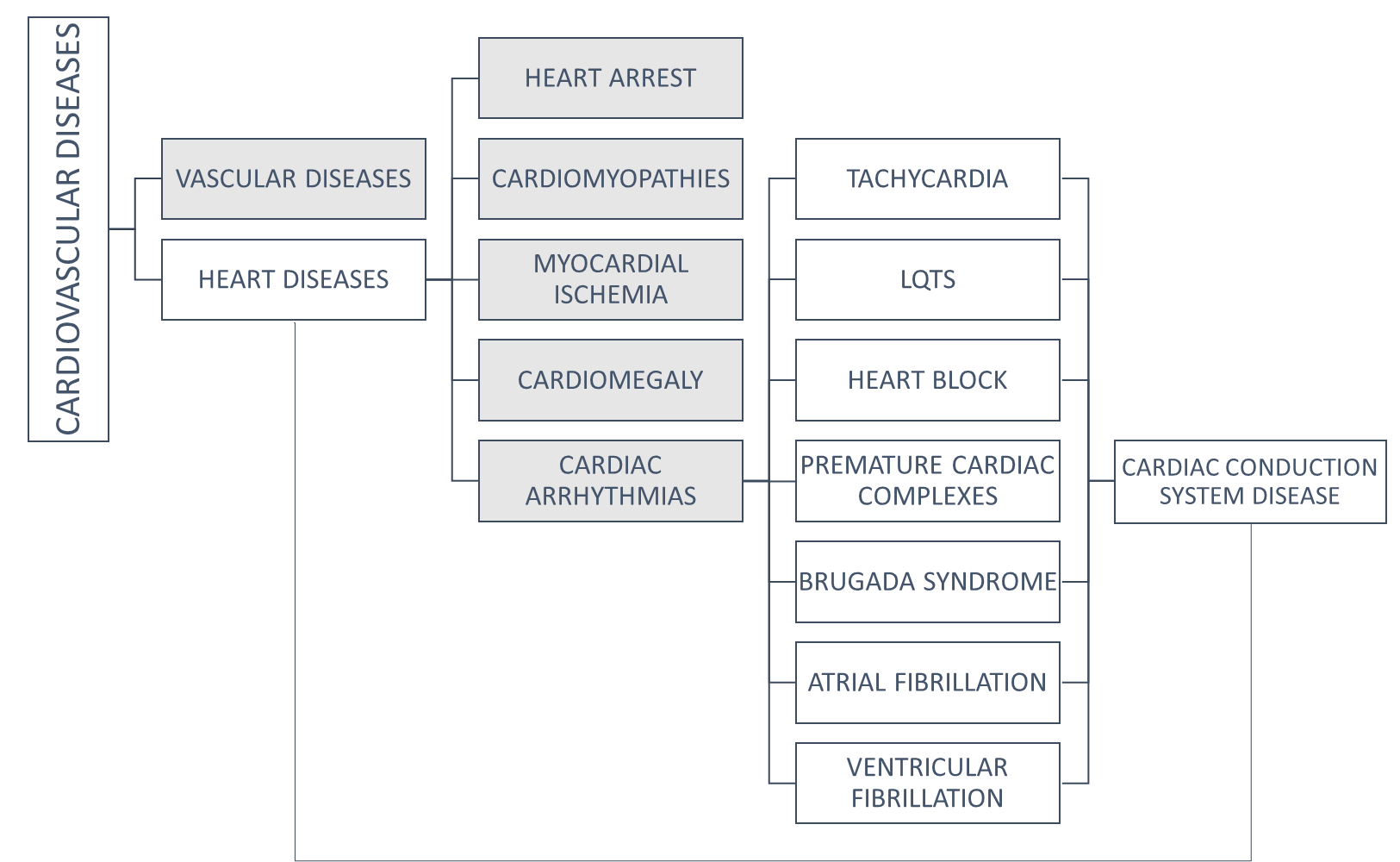
^

**Figure 3.2 Representation of the MeSH categories comprising the nervous system diseases associated to genes.** Main categories shared by these genes are selected and highlighted in grey: 1) neurobehavioral manifestations; 2) febrile seizures; 3) epilepsy; 4) headache disorders; 5) neurodegenerative diseases; 5) neuromuscular diseases. These lower-level categories comprise the “MeSH” terminological-based network of genotype-phenotype associations about nervous system diseases.


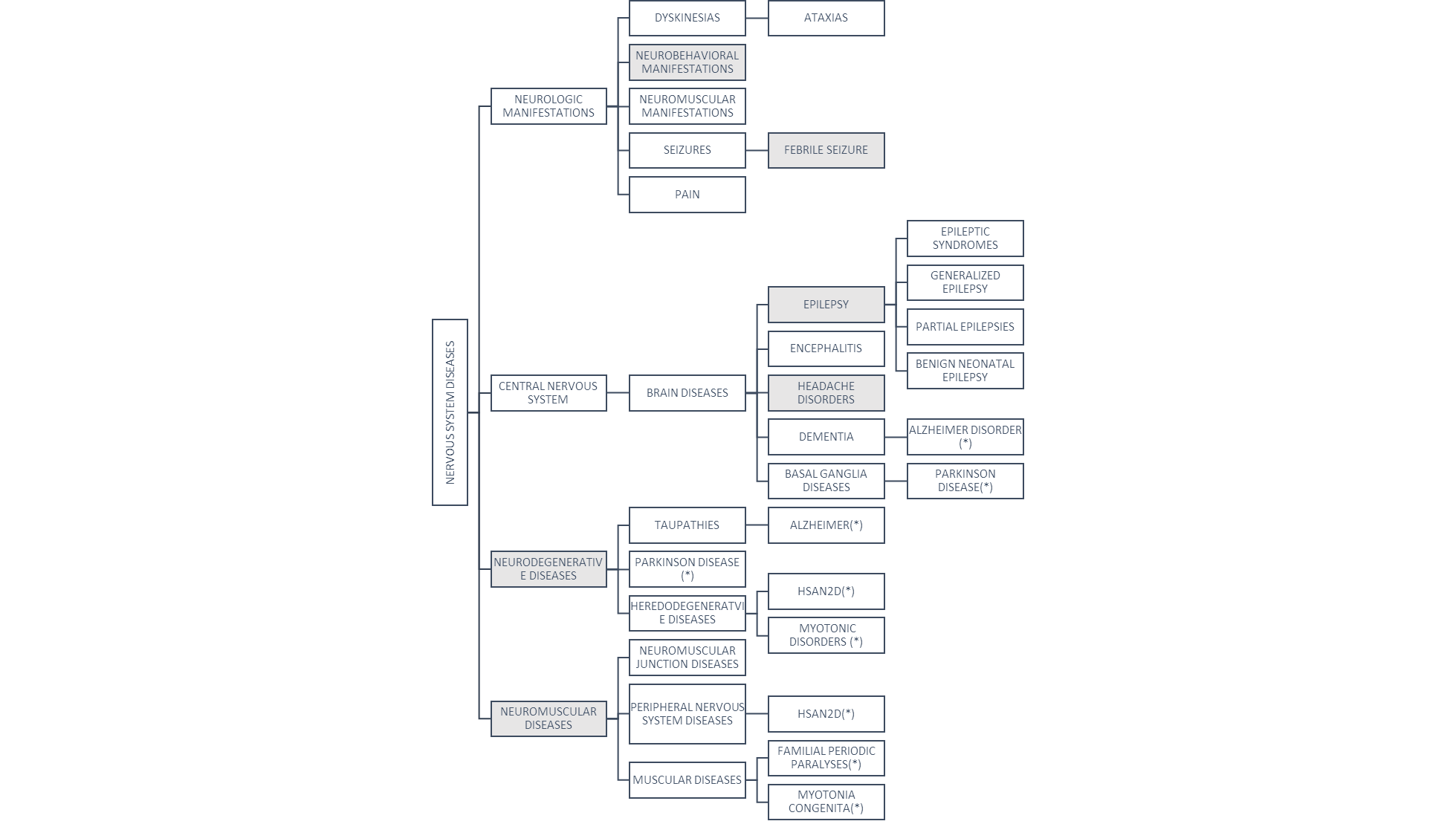


**Figure 3.3 Representation of the MeSH categories comprising the mental disorders associated to genes.** Main categories shared among genes are selected and highlighted in grey: 1) Tobacco use disorder; 2) Other mental disorders which include: bipolar disorder, Alzheimer disease, autism, depression or schizophrenia. These lower-level categories comprise the “MeSH” terminological-based network of genotype-phenotype associations about mental disorders.


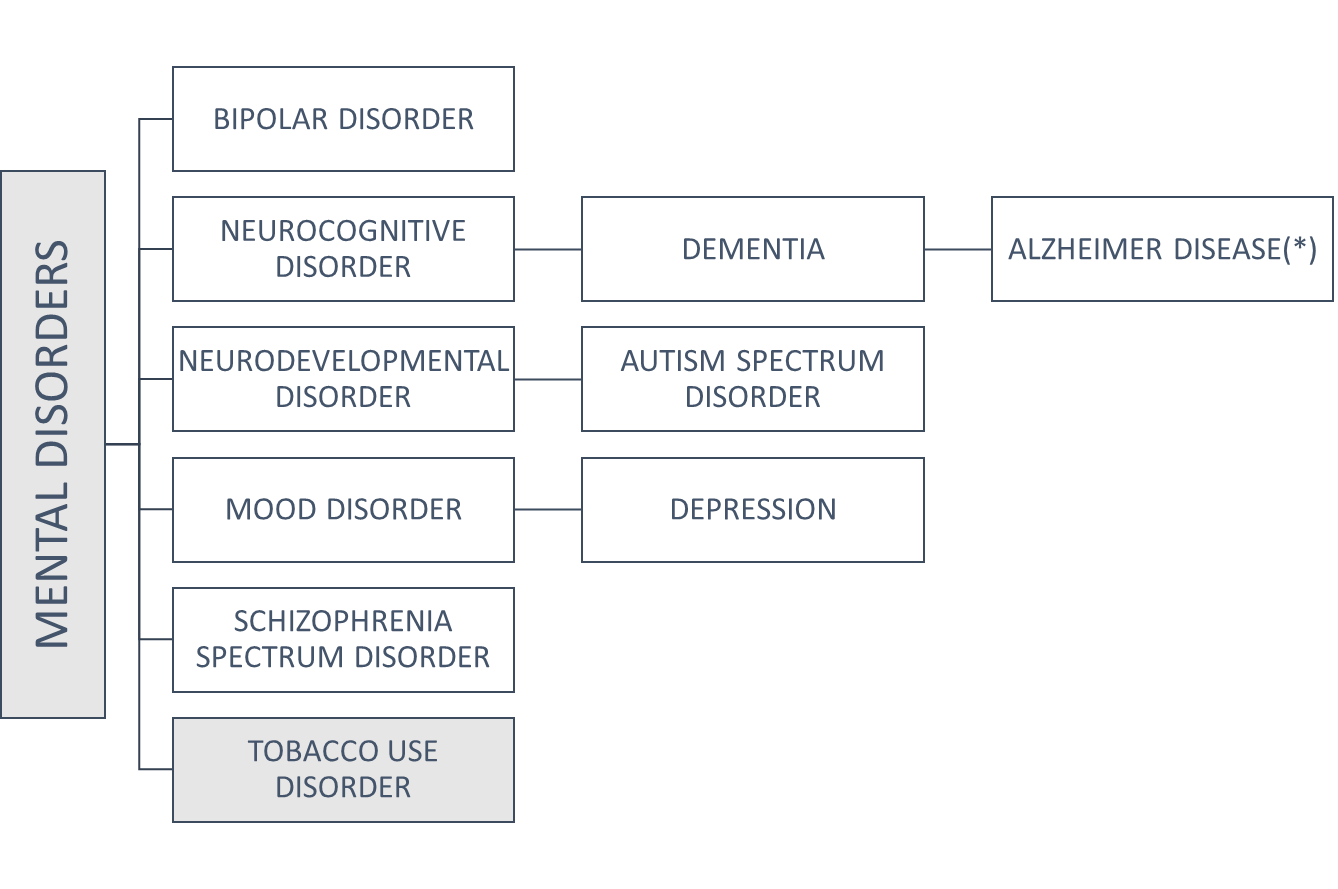


**Figure 3.4 Representation of the MeSH categories comprising the rest of disorders associated to genes.** Main categories shared among genes are selected and highlighted in grey: 1) Sudden death; 2) Diabetes Mellitus type 2; 3) Periodic paralyses. These lower-level categories comprise the “MeSH” terminological-based network of genotype-phenotype associations about these other disorders.


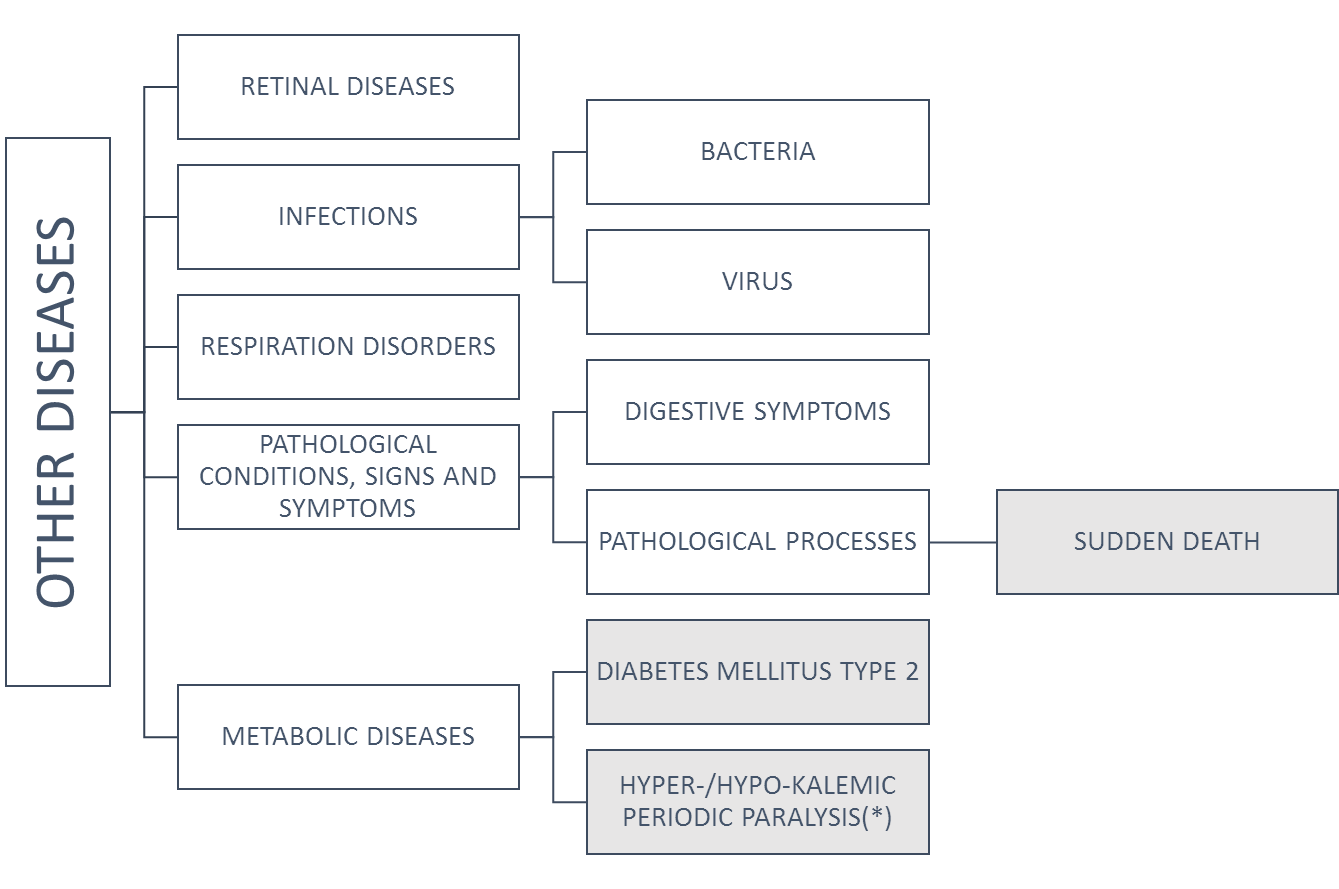

Supplement: Supplementary file 3 — Additional file 3. Description of the upper-level and lower-level categories selected for the creation of “MeSH”-based terminological networks. Hierarchical trees of the upper-level categories (cardiovascular diseases, nervous system diseases, mental diseases, and other diseases) are described in detail. Lower-level categories are based on disease evidences obtained through DAVID search, systematic review and exhaustive review. The categories selected for the genotype-phenotype representations are highlighted in grey. [file 12859_2019_3162_MOESM3_ESM.docx]
