# Supplementary material for: An integrative methodology based on protein-protein interaction networks for identification and functional annotation of disease-relevant genes applied to channelopathies
Source: BMC Bioinformatics. 2019 Nov 12;20:565. doi: 10.1186/s12859-019-3162-1 (PMC6849233; doi:10.1186/s12859-019-3162-1)
Supplement: Supplementary file 4 — Additional file 4. Functional annotation results obtained through Stage 3 of the workflow. Section 1 refers to all the raw functional annotation results of the most relevant genes in channelopathies directly extracted from the last stage of the workflow (DAVID search). Section 2 refers to the extraction of the diseases from this source of information. The diseases then were classified by their lower-level categories according to the “MeSH” criterion described in methods. Some evidences could not be classified due to lack of enough information. These categories will allow the visual representation of genotype-phenotype associations obtained through DAVID search. [file 12859_2019_3162_MOESM4_ESM.docx]

- 1. **Raw functional annotation results directly extracted from the last stage of the workflow (DAVID search).**

**Table 4.1.1 Summary of gene-specific information**. Summary of gene-specific functional annotation of each gene after DAVID automatic search in Entrez Gene database.

| ID | Gene Name | Species | ENTREZ_GENE_SUMMARY |
| --- | --- | --- | --- |
| ANK3 | Ankyrin 3(ANK3) | Homo sapiens | Ankyrins are a family of proteins that are believed to link the integral membrane proteins to the underlying spectrin-actin cytoskeleton and play key roles in activities such as cell motility, activation, proliferation, contact, and the maintenance of specialized membrane domains. Multiple isoforms of ankyrin with different affinities for various target proteins are expressed in a tissue-specific, developmentally regulated manner. Most ankyrins are typically composed of three structural domains: an amino-terminal domain containing multiple ankyrin repeats; a central region with a highly conserved spectrin binding domain; and a carboxy-terminal regulatory domain which is the least conserved and subject to variation. Ankyrin 3 is an immunologically distinct gene product from ankyrins 1 and 2, and was originally found at the axonal initial segment and nodes of Ranvier of neurons in the central and peripheral nervous systems. Multiple transcript variants encoding different isoforms have been found for this gene.[provided by RefSeq, Feb 2011], |
| KCNH2 | Potassium voltage-gated channel subfamily H member 2(KCNH2) | Homo sapiens | This gene encodes a voltage-activated potassium channel belonging to the eag family. It shares sequence similarity with the Drosophila ether-a-go-go (eag) gene. Mutations in this gene can cause long QT syndrome type 2 (LQT2). Transcript variants encoding distinct isoforms have been identified. [provided by RefSeq, Jul 2008], |
| KCNQ2 | Potassium voltage-gated channel subfamily Q member 2(KCNQ2) | Homo sapiens | The M channel is a slowly activating and deactivating potassium channel that plays a critical role in the regulation of neuronal excitability. The M channel is formed by the association of the protein encoded by this gene and a related protein encoded by the KCNQ3 gene, both integral membrane proteins. M channel currents are inhibited by M1 muscarinic acetylcholine receptors and activated by retigabine, a novel anti-convulsant drug. Defects in this gene are a cause of benign familial neonatal convulsions type 1 (BFNC), also known as epilepsy, benign neonatal type 1 (EBN1). At least five transcript variants encoding five different isoforms have been found for this gene. [provided by RefSeq, Jul 2008], |
| SCN1A | Sodium voltage-gated channel alpha subunit 1(SCN1A) | Homo sapiens | Voltage-dependent sodium channels are heteromeric complexes that regulate sodium exchange between intracellular and extracellular spaces and are essential for the generation and propagation of action potentials in muscle cells and neurons. Each sodium channel is composed of a large pore-forming, glycosylated alpha subunit and two smaller beta subunits. This gene encodes a sodium channel alpha subunit, which has four homologous domains, each of which contains six transmembrane regions. Allelic variants of this gene are associated with generalized epilepsy with febrile seizures and epileptic encephalopathy. Alternative splicing results in multiple transcript variants. The RefSeq Project has decided to create four representative RefSeq records. Three of the transcript variants are supported by experimental evidence and the fourth contains alternate 5&apos; untranslated exons, the exact combination of which have not been experimentally confirmed for the full-length transcript. [provided by RefSeq, Oct 2015], |
| SCN2A | sodium voltage-gated channel alpha subunit 2(SCN2A) | Homo sapiens | Voltage-gated sodium channels are transmembrane glycoprotein complexes composed of a large alpha subunit with 24 transmembrane domains and one or more regulatory beta subunits. They are responsible for the generation and propagation of action potentials in neurons and muscle. This gene encodes one member of the sodium channel alpha subunit gene family. It is heterogeneously expressed in the brain, and mutations in this gene have been linked to several seizure disorders. Several alternatively spliced transcript variants of this gene have been described, but the full-length nature of some of these variants has not been determined. [provided by RefSeq, Jul 2008], |
| SCN4A | sodium voltage-gated channel alpha subunit 4(SCN4A) | Homo sapiens | Voltage-gated sodium channels are transmembrane glycoprotein complexes composed of a large alpha subunit with 24 transmembrane domains and one or more regulatory beta subunits. They are responsible for the generation and propagation of action potentials in neurons and muscle. This gene encodes one member of the sodium channel alpha subunit gene family. It is expressed in skeletal muscle, and mutations in this gene have been linked to several myotonia and periodic paralysis disorders. [provided by RefSeq, Jul 2008], |
| SCN5A | sodium voltage-gated channel alpha subunit 5(SCN5A) | Homo sapiens | The protein encoded by this gene is an integral membrane protein and tetrodotoxin-resistant voltage-gated sodium channel subunit. This protein is found primarily in cardiac muscle and is responsible for the initial upstroke of the action potential in an electrocardiogram. Defects in this gene are a cause of long QT syndrome type 3 (LQT3), an autosomal dominant cardiac disease. Alternative splicing results in several transcript variants encoding different isoforms. [provided by RefSeq, Jul 2008], |
| SCN9A | sodium voltage-gated channel alpha subunit 9(SCN9A) | Homo sapiens | This gene encodes a voltage-gated sodium channel which plays a significant role in nociception signaling. Mutations in this gene have been associated with primary erythermalgia, channelopathy-associated insensitivity to pain, and paroxysmal extreme pain disorder. [provided by RefSeq, Aug 2009], |
| SCN4B | sodium voltage-gated channel beta subunit 4(SCN4B) | Homo sapiens | The protein encoded by this gene is one of several sodium channel beta subunits. These subunits interact with voltage-gated alpha subunits to change sodium channel kinetics. The encoded transmembrane protein forms interchain disulfide bonds with SCN2A. Defects in this gene are a cause of long QT syndrome type 10 (LQT10). Three protein-coding and one non-coding transcript variant have been found for this gene.[provided by RefSeq, Mar 2009], |

**Table 4.1.2**. **Accession numbers to Ensembl of the genes of interest**. The accession numbers of each gene to Ensembl tool after DAVID automatic search.

| ID | Gene Name | Species | ENSEMBL_GENE_ID |
| --- | --- | --- | --- |
| ANK3 | ankyrin 3 | Homo sapiens | ENSG00000151150 |
| KCNH2 | potassium voltage-gated channel subfamily H member 2 | Homo sapiens | ENSG00000055118 |
| KCNQ2 | potassium voltage-gated channel subfamily Q member 2 | Homo sapiens | ENSG00000075043 |
| SCN1A | sodium voltage-gated channel alpha subunit 1 | Homo sapiens | ENSG00000144285 |
| SCN2A | sodium voltage-gated channel alpha subunit 2 | Homo sapiens | ENSG00000136531 |
| SCN4A | sodium voltage-gated channel alpha subunit 4 | Homo sapiens | ENSG00000007314 |
| SCN5A | sodium voltage-gated channel alpha subunit 5 | Homo sapiens | ENSG00000183873 |
| SCN9A | sodium voltage-gated channel alpha subunit 9 | Homo sapiens | ENSG00000169432 |
| SCN4B | sodium voltage-gated channel beta subunit 4 | Homo sapiens | ENSG00000177098 |

**Table 4.1.3.** **Gene localization and subcellular distribution**. Localization of each gene of interest and their subcellular distribution after DAVID automatic search in Sequence annotation of Uniprot database*.*

| ID | Gene Name | Species | UP_SEQ_FEATURE |
| --- | --- | --- | --- |
| ANK3 | ankyrin 3 | Homo sapiens | chain:Ankyrin-3,compositionally biased region:Poly-Asn,compositionally biased region:Poly-Glu,compositionally biased region:Poly-Pro,compositionally biased region:Poly-Ser,compositionally biased region:Poly-Thr,compositionally biased region:Ser-rich,compositionally biased region:Thr-rich,cross-link:Glycyl lysine isopeptide (Lys-Gly) (interchain with G-Cter in ubiquitin),domain:Death,domain:ZU5,modified residue,repeat:ANK 1,repeat:ANK 10,repeat:ANK 11,repeat:ANK 12,repeat:ANK 13,repeat:ANK 14,repeat:ANK 15,repeat:ANK 16,repeat:ANK 17,repeat:ANK 18,repeat:ANK 19,repeat:ANK 2,repeat:ANK 20,repeat:ANK 21,repeat:ANK 22,repeat:ANK 23,repeat:ANK 3,repeat:ANK 4,repeat:ANK 5,repeat:ANK 6,repeat:ANK 7,repeat:ANK 8,repeat:ANK 9,sequence conflict,sequence variant |
| KCNH2 | potassium voltage-gated channel subfamily H member 2 | Homo sapiens | chain:Potassium voltage-gated channel subfamily H member 2,compositionally biased region:Poly-Pro,domain:PAC,domain:PAS,glycosylation site:N-linked (GlcNAc...),helix,modified residue,mutagenesis site,nucleotide phosphate-binding region:cNMP,region of interest:Segment H5 (pore-forming),sequence variant,short sequence motif:Selectivity filter,splice variant,strand,topological domain:Cytoplasmic,transmembrane region |
| KCNQ2 | potassium voltage-gated channel subfamily Q member 2 | Homo sapiens | chain:Potassium voltage-gated channel subfamily KQT member 2,modified residue,mutagenesis site,region of interest:Segment H5 (pore-forming),sequence conflict,sequence variant,short sequence motif:Selectivity filter,splice variant,transmembrane region |
| SCN1A | sodium voltage-gated channel alpha subunit 1 | Homo sapiens | chain:Sodium channel protein type 1 subunit alpha,glycosylation site:N-linked (GlcNAc...),repeat:I,repeat:II,repeat:III,repeat:IV,sequence conflict,sequence variant,splice variant,transmembrane region |
| SCN2A | sodium voltage-gated channel alpha subunit 2 | Homo sapiens | chain:Sodium channel protein type 2 subunit alpha,domain:IQ,glycosylation site:N-linked (GlcNAc...),helix,repeat:I,repeat:II,repeat:III,repeat:IV,sequence conflict,sequence variant,splice variant,strand,transmembrane region |
| SCN4A | sodium voltage-gated channel alpha subunit 4 | Homo sapiens | chain:Sodium channel protein type 4 subunit alpha,domain:IQ,glycosylation site:N-linked (GlcNAc...),repeat:I,repeat:II,repeat:III,repeat:IV,sequence conflict,sequence variant,transmembrane region |
| SCN5A | sodium voltage-gated channel alpha subunit 5 | Homo sapiens | chain:Sodium channel protein type 5 subunit alpha,domain:IQ,glycosylation site:N-linked (GlcNAc...),helix,mutagenesis site,region of interest:Interaction with NEDD4, NEDD4L and WWP2,sequence conflict,sequence variant,splice variant,strand,transmembrane region,turn |
| SCN9A | sodium voltage-gated channel alpha subunit 9 | Homo sapiens | chain:Sodium channel protein type 9 subunit alpha,domain:IQ,glycosylation site:N-linked (GlcNAc...),modified residue,repeat:I,repeat:II,repeat:III,repeat:IV,sequence conflict,sequence variant,splice variant,transmembrane region |
| SCN4B | sodium voltage-gated channel beta subunit 4 | Homo sapiens | chain:Sodium channel subunit beta-4,disulfide bond,domain:Ig-like C2-type,glycosylation site:N-linked (GlcNAc...),sequence conflict,sequence variant,signal peptide,splice variant,topological domain:Cytoplasmic,topological domain:Extracellular,transmembrane region |

**Table 4.1.4.** **Tissue specificities of the genes.** Human tissue expressions of the genes after DAVID automatic search in Tissue specificity of Uniprot database.

| ID | Gene Name | Species | UP_TISSUE |
| --- | --- | --- | --- |
| ANK3 | ankyrin 3 | Homo sapiens | Amygdala, Brain, Brain stem, Cervix, Fetal kidney, Kidney, Placenta |
| KCNH2 | potassium voltage-gated channel subfamily H member 2 | Homo sapiens | Brain, Heart, Heart atrium, Heart ventricle, Hippocampus |
| KCNQ2 | potassium voltage-gated channel subfamily Q member 2 | Homo sapiens | Brain, Eye, Fetal brain, Neuroblastoma, Temporal cortex |
| SCN1A | sodium voltage-gated channel alpha subunit 1 | Homo sapiens | Brain, Normal brain |
| SCN2A | sodium voltage-gated channel alpha subunit 2 | Homo sapiens | Brain |
| SCN4A | sodium voltage-gated channel alpha subunit 4 | Homo sapiens | Skeletal muscle |
| SCN5A | sodium voltage-gated channel alpha subunit 5 | Homo sapiens | Brain, Breast, Heart, Jejunal smooth muscle, Ovary |
| SCN9A | sodium voltage-gated channel alpha subunit 9 | Homo sapiens | Epithelium, Spinal ganglion, Thyroid |
| SCN4B | sodium voltage-gated channel beta subunit 4 | Homo sapiens | Brain, Placenta |

**Table 4.1.5.** **Summary of gene functions.** Gene functions obtained after DAVID automatic search in the Protein Information Resource (PIR) database*.*

| ID | Gene Name | Species | PIR_SUMMARY |
| --- | --- | --- | --- |
| ANK3 | ankyrin 3 | Homo sapiens | Membrane-cytoskeleton linker. The neural-specific isoforms may participate in the maintenance/targeting of ion channels and cell adhesion molecules at the nodes of Ranvier and axonal initial segments. |
| KCNH2 | potassium voltage-gated channel subfamily H member 2 | Homo sapiens | Pore-forming (alpha) subunit of voltage-gated inwardly rectifying potassium channel. Channel properties are modulated by cAMP and subunit assembly. Mediates the rapidly activating component of the delayed rectifying potassium current in heart (IKr). Isoform 3 has no channel activity by itself, but modulates channel characteristics when associated with isoform 1. |
| SCN1A | sodium voltage-gated channel alpha subunit 1 | Homo sapiens | Mediates the voltage-dependent sodium ion permeability of excitable membranes. Assuming opened or closed conformations in response to the voltage difference across the membrane, the protein forms a sodium-selective channel through which Na(+) ions may pass in accordance with their electrochemical gradient. |
| SCN2A | sodium voltage-gated channel alpha subunit 2 | Homo sapiens | Mediates the voltage-dependent sodium ion permeability of excitable membranes. Assuming opened or closed conformations in response to the voltage difference across the membrane, the protein forms a sodium-selective channel through which Na(+) ions may pass in accordance with their electrochemical gradient. |
| SCN4A | sodium voltage-gated channel alpha subunit 4 | Homo sapiens | This protein mediates the voltage-dependent sodium ion permeability of excitable membranes. Assuming opened or closed conformations in response to the voltage difference across the membrane, the protein forms a sodium-selective channel through which Na(+) ions may pass in accordance with their electrochemical gradient. This sodium channel may be present in both denervated and innervated skeletal muscle. |
| SCN4B | sodium voltage-gated channel beta subunit 4 | Homo sapiens | Modulates channel gating kinetics. Causes negative shifts in the voltage dependence of activation of certain alpha sodium channels, but does not affect the voltage dependence of inactivation (By similarity). |

**Table 4.1.6.** **Accession numbers of Reactome pathways.** Accession numbers of the interactome pathways where the genes of interest are involved after DAVID functional annotation in Reactome database.

| ID | Gene Name | Species | REACTOME_PATHWAY |
| --- | --- | --- | --- |
| ANK3 | ankyrin 3 | Homo sapiens | R-HSA-445095:R-HSA-445095, R-HSA-6807878:R-HSA-6807878 |
| KCNH2 | potassium voltage-gated channel subfamily H member 2 | Homo sapiens | R-HSA-1296072:R-HSA-1296072,R-HSA-5576890:R-HSA-5576890 |
| KCNQ2 | potassium voltage-gated channel subfamily Q member 2 | Homo sapiens | R-HSA-1296072:R-HSA-1296072,R-HSA-445095:R-HSA-445095 |
| SCN1A | sodium voltage-gated channel alpha subunit 1 | Homo sapiens | R-HSA-445095:R-HSA-445095,R-HSA-5576892:R-HSA-5576892 |
| SCN2A | sodium voltage-gated channel alpha subunit 2 | Homo sapiens | R-HSA-445095:R-HSA-445095,R-HSA-5576892:R-HSA-5576892 |
| SCN4A | sodium voltage-gated channel alpha subunit 4 | Homo sapiens | R-HSA-445095:R-HSA-445095,R-HSA-5576892:R-HSA-5576892 |
| SCN5A | sodium voltage-gated channel alpha subunit 5 | Homo sapiens | R-HSA-445095:R-HSA-445095,R-HSA-5576892:R-HSA-5576892 |
| SCN9A | sodium voltage-gated channel alpha subunit 9 | Homo sapiens | R-HSA-445095:R-HSA-445095,R-HSA-5576892:R-HSA-5576892 |
| SCN4B | sodium voltage-gated channel beta subunit 4 | Homo sapiens | R-HSA-445095:R-HSA-445095,R-HSA-5576892:R-HSA-5576892 |

**Table 4.1.7. Diseases classes evidenced in the genes of interest.** Classes on which the evidences of the diseases are classified after the functional annotation by DAVID in Genetic Association Database (GAD).

| ID | Gene Name | Species | GAD_DISEASE_CLASS |
| --- | --- | --- | --- |
| ANK3 | ankyrin 3 | Homo sapiens | Cardiovascular, Chemdependency, Metabolic, Neurological, Psych, Renal |
| KCNH2 | potassium voltage-gated channel subfamily H member 2 | Homo sapiens | Cardiovascular, Chemdependency, Metabolic, Other, Pharmacogenomic, Psych, Unknown |
| KCNQ2 | potassium voltage-gated channel subfamily Q member 2 | Homo sapiens | Neurological |
| SCN1A | sodium voltage-gated channel alpha subunit 1 | Homo sapiens | Cardiovascular, Metabolic, Neurological, Other, Pharmacogenomic, Unknown |
| SCN2A | sodium voltage-gated channel alpha subunit 2 | Homo sapiens | Cardiovascular, Chemdependency, Neurological, Psych, Unknown |
| SCN4A | sodium voltage-gated channel alpha subunit 4 | Homo sapiens | Other, Pharmacogenomic, Unknown, Vision |
| SCN5A | sodium voltage-gated channel alpha subunit 5 | Homo sapiens | Cardiovascular, Chemdependency, Immune, Infection, Metabolic, Neurological, Other, Pharmacogenomic, Unknown |
| SCN9A | sodium voltage-gated channel alpha subunit 9 | Homo sapiens | Chemdependency, Developmental, Metabolic, Neurological, Psych |
| SCN4B | sodium voltage-gated channel beta subunit 4 | Homo sapiens | Cardiovascular |

**Table 4.1.8**. **Diseases evidenced in each gene of interest.** Evidences of the diseases found as functional annotation results after DAVID search in Genetic Association Database (GAD) and Online Mendelian Inheritance in Man database (OMIM).

| ID | Gene Name | Species | DATABASE | DISEASE |
| --- | --- | --- | --- | --- |
| ANK3 | ankyrin 3 | Homo sapiens | GAD | Alzheimer's Disease,Alzheimer's disease ,Arteries,Bipolar Disorder,Cholesterol, LDL,Creatinine,Glomerular Filtration Rate,Schizophrenia,Tobacco Use Disorder,Triglycerides |
|  |  |  | OMIM | 615493~Mental retardation, autosomal recessive, 37 |
| KCNH2 | potassium voltage-gated channel subfamily H member 2 | Homo sapiens | GAD | Arrhythmias, Cardiac\|,Arrhythmias, Cardiac\|Brugada Syndrome\|Cardiomyopathies\|Channelopathies\|Death, Sudden, Cardiac\|Sudden Cardiac Death,Arrhythmias, Cardiac\|Death, Sudden, Cardiac\|,Arrhythmias, Cardiac\|Heart Arrest\|Long QT Syndrome\|Syncope,Arrhythmias, Cardiac\|Long QT Syndrome,Atrial Fibrillation,Atrial Fibrillation\|,Atrial Fibrillation\|Hypertension,Atrioventricular Block\|Death, Sudden\|Syncope\|Torsades de Pointes,atriventricular block long QT syndrome,Brugada Syndrome\|Chromosome Deletion\|Death, Sudden, Cardiac\|Long QT Syndrome\|Sudden Cardiac Death,cardiac repolarization,cardiac repolarization.,cardiovascular,cardiovascular disease,Death, Sudden, Cardiac\|Long QT Syndrome,Death, Sudden, Cardiac\|Long QT Syndrome\|Sudden Cardiac Death,Death, Sudden, Cardiac\|Sudden Cardiac Death,depression \| long QT syndrome,Diabetes Mellitus, Type 2\|Long QT Syndrome,EKG, abnormal,EKG, abnormal; Brugada syndrome,Electrocardiographic traits ,Electrocardiography,gastrointestinal symptoms,Gastroparesis,Long QT Syndrome,Long QT Syndrome\|Sinus Tachycardia\|Tachycardia, Sinus,Long QT Syndrome\|Sudden Infant Death,Long QT Syndrome\|Torsades de Pointes,long-QT syndrome,null,pharmacogenetic studies,QT interval,Schizophrenia,SIDS/sudden infant death syndrome,Sudden Infant Death,Type 2 Diabetes\| edema \| rosiglitazone,unknown |
|  |  |  | OMIM | 609620~Short QT syndrome 1,613688~Long QT syndrome 2,613688~Long QT syndrome 2, acquired, susceptibility to |
| KCNQ2 | potassium voltage-gated channel subfamily Q member 2 | Homo sapiens | GAD | epilepsy,Epilepsy\|Syndrome |
|  |  |  | OMIM | 121200~Myokymia,121200~Seizures, benign neonatal, 1,613720~Epileptic encephalopathy, early infantile, 7 |
| SCN1A | sodium voltage-gated channel alpha subunit 1 | Homo sapiens | GAD | Blood Pressure,Electrocardiography,Epilepsies, Myoclonic,Epilepsies, Partial\|Epilepsy, Generalized\|Seizures, Febrile,epilepsy,Epilepsy, Generalized\|Seizures, Febrile,Epilepsy\|,generalized epilepsy,Hip,null,phenytoin,seizures,Seizures, Febrile\|Syndrome,severe myoclonic epilepsy of infancy |
|  |  |  | OMIM | 604403~Epilepsy, generalized, with febrile seizures plus, type 2,604403~Febrile seizures, familial, 3A,607208~Dravet syndrome,609634~Migraine, familial hemiplegic, 3 |
| SCN2A | sodium voltage-gated channel alpha subunit 2 | Homo sapiens | GAD | autism,Coronary Artery Disease,epilepsy,Epilepsy, Generalized,Epilepsy, Generalized\|Seizures,Epilepsy\|,null,Parkinson Disease,Peripheral Nervous System Diseases,seizures, febrile,Tobacco Use Disorder |
|  |  |  | OMIM | 607745~Seizures, benign familial infantile, 3,613721~Epileptic encephalopathy, early infantile, 11 |
| SCN4A | sodium voltage-gated channel alpha subunit 4 | Homo sapiens | GAD | benzene haematotoxicity,macular degeneration,Myotonia Congenita,thyrotoxic periodic paralysis |
|  |  |  | OMIM | 168300~Paramyotonia congenita,170500~Hyperkalemic periodic paralysis, type 2,608390~Myotonia congenita, atypical, acetazolamide-responsive,613345~Hypokalemic periodic paralysis, type 2,614198~Myasthenic syndrome, congenital, 16 |
| SCN5A | sodium voltage-gated channel alpha subunit 5 | Homo sapiens | GAD | arrhythmia, cardiac,Arrhythmias, Cardiac\|,Arrhythmias, Cardiac\|Brugada Syndrome\|Cardiomyopathies\|Channelopathies\|Death, Sudden, Cardiac\|Sudden Cardiac Death,Arrhythmias, Cardiac\|Brugada Syndrome\|Death, Sudden, Cardiac,Arrhythmias, Cardiac\|Cardiovascular Diseases,Arrhythmias, Cardiac\|Death, Sudden, Cardiac\|,Arrhythmias, Cardiac\|Heart Failure\|Hypertrophy, Left Ventricular,Arrhythmias, Cardiac\|Long QT Syndrome,Arrhythmias, Cardiac\|Myocardial Infarction,Arrhythmias, Cardiac\|Sudden Infant Death,Atrial Fibrillation,Atrial Fibrillation\|,Atrial Fibrillation\|Brugada Syndrome,Atrial Fibrillation\|Brugada Syndrome\|Tachycardia, Ventricular,Atrial Fibrillation\|Heart Diseases,Atrioventricular Block\|Death, Sudden\|Syncope\|Torsades de Pointes,atriventricular block long QT syndrome,Brugada Syndrome,Brugada Syndrome\|,Brugada Syndrome\|Chromosome Deletion\|Death, Sudden, Cardiac\|Long QT Syndrome\|Sudden Cardiac Death,Brugada Syndrome\|Death, Sudden, Cardiac\|Sudden Cardiac Death,Brugada Syndrome\|Recurrence\|Shock\|Ventricular Fibrillation,Brugada Syndrome\|Ventricular Fibrillation\|Ventricular Premature Complexes,cardiac arrhythmias and sudden death,cardiac conduction disturbances and degenerative changes,cardiac death,Cardiomyopathy, Dilated\|DCM - Dilated cardiomyopathy,Cardiovascular Diseases,Congenital sick sinus syndrome,Death, Sudden,Death, Sudden, Cardiac\|Sudden Cardiac Death,Death, Sudden\|Sudden Infant Death,depression \| long QT syndrome,Diabetes Mellitus, Type 2\|Long QT Syndrome,diabetes, type 1 ,dilated cardiomyopathy,Drug-induced long-QT syndrome,early onset of sudden infant death.,EKG, abnormal,Electrocardiographic conduction measures,Electrocardiography,gastrointestinal symptoms,Heart Function Tests,HIV tuberculosis,inherited cardiac arrhythmia long QT syndrome,Irritable Bowel Syndrome,Long QT Syndrome,Long QT Syndrome\|Sinus Tachycardia\|Tachycardia, Sinus,Long QT Syndrome\|Sudden Infant Death,long-QT syndrome,Migraine without Aura,myocardial infarct,Myocardial Infarction\|Ventricular Fibrillation,null,PR interval,QT interval,resting heart rate,SIDS/sudden infant death syndrome,Sudden Infant Death,thyrotoxic periodic paralysis,Tobacco Use Disorder,Type 2 Diabetes\| edema \| rosiglitazone |
|  |  |  | OMIM | 113900~Heart block, nonprogressive,113900~Heart block, progressive, type IA,272120~Sudden infant death syndrome, susceptibility to,601144~Brugada syndrome 1,601154~Cardiomyopathy, dilated, 1E,603829~Ventricular fibrillation, familial, 1,603830~Long QT syndrome-3,608567~Sick sinus syndrome 1,614022~Atrial fibrillation, familial, 10 |
| SCN9A | sodium voltage-gated channel alpha subunit 9 | Homo sapiens | GAD | Abnormalities, Multiple\|Epilepsy\|Seizures, Febrile\|Syndrome,Insulin,Migraine without Aura,Pain,personality,Tobacco Use Disorder |
|  |  |  | OMIM | 133020~Erythermalgia, primary,133020~Small fiber neuropathy,243000~HSAN2D, autosomal recessive,243000~Insensitivity to pain, congenital,607208~Dravet syndrome, modifier of,613863~Epilepsy, generalized, with febrile seizures plus, type 7,613863~Febrile seizures, familial, 3B,Paroxysmal extreme pain disorder, 167400,~Paroxysmal extreme pain disorder, 167400 |
| SCN4B | sodium voltage-gated channel beta subunit 4 | Homo sapiens | GAD | Atrial Fibrillation\| |
|  |  |  | OMIM | 611819~Atrial fibrillation, familial, 17,611819~Long QT syndrome-10 |

**4.2 Dataset of the genotype-phenotype relationships hosted in DAVID database.**

**Table 4.2.1** **Diseases associated with the nine relevant genes using DAVID and classified by the MeSH criterion**. The diseases are classified by lower-level categories according to the MeSH criterion as described in methods. Some evidences could not be classified due to lack of information.

| **DAVID search** | | | |
| --- | --- | --- | --- |
| **Gene** | **Database** | **Disease** | **Lower-level categories** |
| SCN1A | OMIM | Generalized epilepsy with febrile seizures plus type 2 | Febrile seizures / generalized epilepsy |
|  | OMIM | Early infantile epileptic encephalopathy type 6 or Dravet syndrome | Generalized epilepsies / Epileptic syndromes |
|  | OMIM | Familial febrile seizure type 3A | Febrile seizures |
|  | OMIM | Familial hemiplegic migraine type 3 | Headache disorders |
|  | GAD DISEASE | Myoclonic epilepsies | Generalized epilepsy / Epileptic syndromes |
|  | GAD DISEASE | partial epilepsies | Partial epilepsies |
|  | GAD DISEASE | Febrile generalized epilepsies | Febrile seizures / generalized epilepsy |
|  | GAD DISEASE | seizures | Neurologic manifestations |
|  | GAD DISEASE | severe myoclonic epilepsy of infancy | Generalized epilepsies / Epileptic syndromes |
| SCN2A | OMIM | Early infantile epileptic encephalopathy type 11 | Generalized epilepsies / Epileptic syndromes |
|  | OMIM | Benign Familial Infantile Seizures | Benign neonatal Epilepsy |
|  | GAD DISEASE | Autism | Mental disorders |
|  | GAD DISEASE | coronary artery disease | Myocardial ischemia / vascular disease |
|  | GAD DISEASE | generalized seizures | Generalized epilepsy |
|  | GAD DISEASE | epilepsy | Epilepsy |
|  | GAD DISEASE | Parkinson disease | Neurodegenerative diseases / Brain diseases |
|  | GAD DISEASE | Peripheral Nervous System Diseases | Peripheral nervous system diseases |
|  | GAD DISEASE | febrile seizures | Febrile Seizures |
|  | GAD DISEASE | tobacco use disorder | tobacco use disorder |
| SCN4A | OMIM | Hyperkalemic periodic paralysis type 2 | Musculoskeletal diseases / Neuromuscular diseases / Metabolic diseases |
|  | OMIM | Hypokalemic periodic paralysis type 2 | Musculoskeletal diseases / Neuromuscular diseases / Metabolic diseases |
|  | OMIM | Congenital Myasthenic syndrome 16 | Neuromuscular junction diseases |
|  | OMIM | Myotonia congenita atypical | Musculoskeletal diseases / Neurodegenerative diseases / Neuromuscular diseases |
|  | OMIM | Paramyotonia congenital | Musculoskeletal diseases / Neuromuscular diseases |
|  | GAD DISEASE | macular degeneration | Retinal diseases |
|  | GAD DISEASE | myotonia congenita | Musculoskeletal diseases / Neurodegenerative diseases / Neuromuscular disease |
|  | GAD DISEASE | thyrotoxic periodic paralysis | Musculoskeletal diseases / Neuromuscular diseases / Metabolic diseases |
| SCN4B | OMIM | Familial atrial fibrillation type 17 | Cardiac arrhythmias |
|  | OMIM | Long QT syndrome type 10 | Cardiac arrhythmias / Cardiac conduction system disease |
|  | GAD DISEASE | atrial fibrillation | Cardiac arrhythmias |
| SCN5A | OMIM | Familial atrial fibrillation type 10 | Cardiac arrhythmias |
|  | OMIM | Brugada Syndrome type 1 | Cardiac arrhythmias / Cardiac conduction system disease |
|  | OMIM | Cardiomyopathy dilated 1E | Cardiomyopathies |
|  | OMIM | heart block nonprogressive | Cardiac arrhythmias / Cardiac conduction system disease |
|  | OMIM | heart block progressive1A | Cardiac arrhythmias / Cardiac conduction system disease |
|  | OMIM | Long QT syndrome type 3 | Cardiac arrhythmias / Cardiac conduction system disease |
|  | OMIM | Sick sinus syndrome 1 | Cardiac arrhythmias / Cardiac conduction system disease |
|  | OMIM | Familial ventricular fibrillation type 1 | Cardiac arrhythmias |
|  | OMIM | sudden death syndrome | Heart arrest / Pathological Processes |
|  | GAD DISEASE | Cardiac arrhythmia | Cardiac arrhythmias |
|  | GAD DISEASE | brugada syndrome | Cardiac arrhythmias / Cardiac conduction system disease |
|  | GAD DISEASE | cardiomyopathies | Cardiomyopathies |
|  | GAD DISEASE | channelopathies | - |
|  | GAD DISEASE | cardiac sudden death | Heart arrest / Pathological Processes |
|  | GAD DISEASE | heart failure |  |
|  | GAD DISEASE | Left Ventricular hypertrophy | Cardiomegaly |
|  | GAD DISEASE | Long QT syndrome | Cardiac arrhythmias / Cardiac conduction system disease |
|  | GAD DISEASE | Myocardial Infartion | Myocardial ischemia |
|  | GAD DISEASE | Sudden infant death (SIDS) | Pathological processes |
|  | GAD DISEASE | Atrial fibrillation | Cardiac arrhythmias |
|  | GAD DISEASE | tachycardia | Cardiac arrhythmias / Cardiac conduction system disease |
|  | GAD DISEASE | atrioventricular block | Cardiac arrhythmias / Cardiac conduction system disease |
|  | GAD DISEASE | syncope | Neurobehavioral manifestation |
|  | GAD DISEASE | Torsades de Pointes | Cardiac arrhythmias / Cardiac conduction system disease |
|  | GAD DISEASE | cardiac infant death | Heart arrest / Pathological Processes |
|  | GAD DISEASE | ventricular fibrillation | Cardiac arrhythmias |
|  | GAD DISEASE | ventricular premature complexes | Cardiac arrhythmias / Cardiac conduction system disease |
|  | GAD DISEASE | cardiac conduction disturbances and degenerative changes | Cardiac conduction system disease |
|  | GAD DISEASE | dilated cardiomyopathy (DCM) | Cardiomyopathies |
|  | GAD DISEASE | Congenital sick sinus syndrome | Cardiac arrhythmias / Cardiac conduction system disease |
|  | GAD DISEASE | depression | Mental disorder |
|  | GAD DISEASE | Diabetes Mellitus Type 2 | Metabolic diseases |
|  | GAD DISEASE | Drug-induced long-QT syndrome | Cardiac arrhythmias / Cardiac conduction system disease |
|  | GAD DISEASE | early onset of sudden infant death | Pathological processes |
|  | GAD DISEASE | EKG | - |
|  | GAD DISEASE | abnormal Electrocardiographic conduction measures | - |
|  | GAD DISEASE | electrocardiography | - |
|  | GAD DISEASE | gastrointestinal symptoms | Digestive symptoms |
|  | GAD DISEASE | HIV tuberculosis | Infections |
|  | GAD DISEASE | inherited cardiac arrhythmia LQT syndrome | Cardiac arrhythmias / Cardiac conduction system disease |
|  | GAD DISEASE | irritable bowel syndrome | Digestive symptoms |
|  | GAD DISEASE | sinus tachycardia | Cardiac arrhythmias / Cardiac conduction system disease |
|  | GAD DISEASE | migraine without aura | Headache disorders |
|  | GAD DISEASE | thyrotoxic periodic paralysis | Musculoskeletal diseases / Neuromuscular diseases / Metabolic diseases |
|  | GAD DISEASE | tobacco use disorder | tobacco use disorder |
| SCN9A | OMIM | Generalized epilepsy with febrile seizures plus type 7 | Febrile Seizures / Generalized Epilepsy |
|  | OMIM | erythermalgia primary | vascular diseases |
|  | OMIM | Familial Febrile seizures type 3B | febrile seizures |
|  | OMIM | Autosomal recessive congenital indifference to pain (HSAN2D) | peripheral nervous system diseases |
|  | OMIM | insensitivity to pain | peripheral nervous system diseases |
|  | OMIM | paroxysmal extreme pain disorder | peripheral nervous system diseases |
|  | OMIM | small fiber neuropathy | peripheral nervous system diseases |
|  | OMIM | modifier of Dravet syndrome | Generalized epilepsy |
|  | GAD DISEASE | febriles seizures | febriles seizures |
|  | GAD DISEASE | migraine without aura | headache disorders |
|  | GAD DISEASE | tobacco use disorder | Tobacco use disorder |
| KCNQ2 | OMIM | Early infantile epileptic encephalopathy type 7 | Generalized epilepsies / Epileptic syndromes |
|  | OMIM | Myokymia | Neuromuscular manifestations |
|  | OMIM | Benign Neonatal Seizures type 1 | Benign neonatal epilepsy |
|  | GAD DISEASE | Epilepsy syndrome | Epilepsy |
| KCNH2 | OMIM | Long QT syndrome type 2 | Cardiac arrhythmias / Cardiac conduction system disease |
|  | OMIM | Short QT syndrome type 1 | Cardiac arrhythmias / Cardiac conduction system disease |
|  | OMIM | susceptibility to LQTS2 | Cardiac arrhythmias / Cardiac conduction system disease |
|  | GAD DISEASE | cardiac arrhythmias | Cardiac arrhythmias |
|  | GAD DISEASE | brugada syndrome | Cardiac arrhythmias / Cardiac conduction system disease |
|  | GAD DISEASE | Cardiomyopathies | Cardiomyopathies |
|  | GAD DISEASE | Channelopathies | - |
|  | GAD DISEASE | sudden cardiac death | Heart arrest / Pathological Processes |
|  | GAD DISEASE | heart arrest | Heart arrest |
|  | GAD DISEASE | Long QT Syndrome | Cardiac arrhythmias / Cardiac conduction system disease |
|  | GAD DISEASE | syncope | Neurobehavioral manifestation |
|  | GAD DISEASE | atrial fibrillation | Cardiac arrhythmias |
|  | GAD DISEASE | hypertension | vascular diseases |
|  | GAD DISEASE | atrioventricular block | Cardiac arrhythmias / Cardiac conduction system disease |
|  | GAD DISEASE | Torsades de Pointes | Cardiac arrhythmias / Cardiac conduction system disease |
|  | GAD DISEASE | depression | Mental disorder |
|  | GAD DISEASE | diabetes Mellitus type 2 | Metabolic diseases |
|  | GAD DISEASE | EKG abnormal | - |
|  | GAD DISEASE | electrocardiographic traits | - |
|  | GAD DISEASE | gastrointestinal symptoms | Digestive symptoms |
|  | GAD DISEASE | gastroparesis | Digestive symptoms |
|  | GAD DISEASE | sinus tachycardia | Cardiac arrhythmias / Cardiac conduction system disease |
|  | GAD DISEASE | sudden infant death (SIDS) | Heart arrest / Pathological Processes |
|  | GAD DISEASE | schizophrenia | Mental disorders |
| ANK3 | OMIM | mental retardation | Neurobehavioral manifestations |
|  | GAD DISEASE | Alzheimer's Disease | Brain diseases / Neurodegenerative diseases / Mental disorders |
|  | GAD DISEASE | arteries | - |
|  | GAD DISEASE | Bipolar disorder | Mental disorders |
|  | GAD DISEASE | Cholesterol | - |
|  | GAD DISEASE | LDL | - |
|  | GAD DISEASE | Creatinine | - |
|  | GAD DISEASE | Glomerular Filtration Rate | - |
|  | GAD DISEASE | Schizophrenia | Mental disorders |
|  | GAD DISEASE | Tobacco Use Disorder | Tobacco use disorder |
