# Supplementary material for: An integrative methodology based on protein-protein interaction networks for identification and functional annotation of disease-relevant genes applied to channelopathies
Source: BMC Bioinformatics. 2019 Nov 12;20:565. doi: 10.1186/s12859-019-3162-1 (PMC6849233; doi:10.1186/s12859-019-3162-1)
Supplement: Supplementary file 6 — Additional file 6. Dataset of genotype-phenotype relationships found through systematic review of the most relevant genes in channelopathies. Diseases related to the nine relevant genes through the systematic review based on three databases (Gene, OMIM and MedGen). Each phenotype is classified according to its MeSH category, as described in methods. [file 12859_2019_3162_MOESM6_ESM.docx]

**Table 6.1 Dataset of the genotype-phenotype relationships found through systematic review**. Diseases related to the nine relevant genes through the systematic review based on three databases (Gene, OMIM and MedGen). Each phenotype is classified according to its MeSH category.

| **Systematic review** | | | |
| --- | --- | --- | --- |
| **Gene** | **Database** | **Disease** | **Lower-level category** |
| SCN1A | OMIM | Generalized epilepsy with febrile seizures plus type 2 | Febrile seizures / generalized epilepsy |
|  | OMIM | Early infantile epileptic encephalopathy type 6 or Dravet syndrome | Myoclonic epilepsies (Generalized epilepsies / Epileptic syndromes) |
|  | OMIM | Familial febrile seziures type 3A | Febrile seizures |
|  | OMIM | Familial hemiplegic migraine type 3 | Headache disorders |
|  | Gene | Familial hemiplegic migraine type 3 | Headache disorders |
|  | Gene | Generalized epilepsy with febrile seizures plus type 1 | Febrile seizures / generalized epilepsy |
|  | Gene | Generalized epilepsy with febrile seizures plus type 2 | Febrile seizures / generalized epilepsy |
|  | Gene | Severe myoclonic epilepsy of infancy | Myoclonic epilepsies (Generalized epilepsies / Epileptic syndromes) |
|  | MedGen | Generalized epilepsy with febrile seizures plus type 2 | Febrile seizures / generalized epilepsy |
|  | MedGen | Severe myoclonic epilepsy of infancy | Myoclonic epilepsies (Generalized epilepsies / Epileptic syndromes) |
|  | MedGen | Familial hemiplegic migraine type 3 | Headache disorders |
|  | MedGen | Generalized epilepsy with febrile seizures plus type 1 | Febrile seizures / generalized epilepsy |
|  | MedGen | Migraine | Headache disorders |
|  | MedGen | Early infantile epileptic encephalopathy type 1 | Infantile spasms (Generalized epilepsies / Epileptic syndromes) |
|  | MedGen | Familial febrile seizures type 1 | Febrile seizures |
| SCN2A | OMIM | Early infantile epileptic encephalopathy type 11 | Infantile spasms (Generalized epilepsies / Epileptic syndromes) |
|  | OMIM | Benign Familial Infantile Seizures type 3 | Benign neonatal Epilepsy |
|  | Gene | Benign Familial Infantile Seizures | Benign neonatal Epilepsy |
|  | Gene | Early infantile epileptic encephalopathy type 11 | Infantile spasms (Generalized epilepsies / Epileptic syndromes) |
|  | Gene | Generalized epilepsy with febrile seizures plus | Febrile seizures / generalized epilepsy |
|  | MedGen | Benign Familial Neonatal Infantile seizures | Benign neonatal epilepsy |
|  | MedGen | Early infantile epileptic encephalopathy type 11 | Infantile spasms (Generalized epilepsies / Epileptic syndromes) |
|  | MedGen | Early infantile epileptic encephalopathy type 1 | Infantile spasms (Generalized epilepsies / Epileptic syndromes) |
|  | MedGen | Generalized epilepsy with febrile seizures plus | Febrile seizures / generalized epilepsy |
|  | MedGen | Benign Familial Infantile Seizures type 1 | Benign neonatal epilepsy |
| SCN4A | OMIM | Hyperkalemic periodic paralysis type 2 | Musculoskeletal diseases / Neuromuscular diseases / Metabolic diseases |
|  | OMIM | Hypokalemic periodic paralysis type 2 | Musculoskeletal diseases / Neuromuscular diseases / Metabolic diseases |
|  | OMIM | Congenital Myasthenic synrdrome16 | Neuromuscular junction diseases |
|  | OMIM | Myotonia congenita atypical | Musculoskeletal diseases / Neurodegenerative diseases / Neuromuscular diseases |
|  | OMIM | Paramyotonia congenita | Musculoskeletal diseases / Neuromuscular diseases |
|  | Gene | Congenital myasthenic syndrome, acetazolamide-responsive | Neuromuscular junction diseases |
|  | Gene | Hyperkalemic Periodic Paralysis Type 1 | Musculoskeletal diseases / Neuromuscular diseases / Metabolic diseases |
|  | Gene | Hypokalemic periodic paralysis 1 | Musculoskeletal diseases / Neuromuscular diseases / Metabolic diseases |
|  | Gene | Hypokalemic periodic paralysis, type 2 | Musculoskeletal diseases / Neuromuscular diseases / Metabolic diseases |
|  | Gene | Paramyotonia congenita of von Eulenburg | Musculoskeletal diseases / Neuromuscular diseases |
|  | Gene | Potassium aggravated myotonia | Musculoskeletal diseases / Neurodegenerative diseases / Neuromuscular diseases |
|  | MedGen | Paramyotonia congenita of von Eulenburg | Musculoskeletal diseases / Neuromuscular diseases |
|  | MedGen | Hyperkalemic Periodic Paralysis Type 1 | Musculoskeletal diseases / Neuromuscular diseases / Metabolic diseases |
|  | MedGen | Hypokalemic periodic paralysis 1 | Musculoskeletal diseases / Neuromuscular diseases / Metabolic diseases |
|  | MedGen | Hypokalemic periodic paralysis, type 2 | Musculoskeletal diseases / Neuromuscular diseases / Metabolic diseases |
|  | MedGen | Congenital myasthenic syndrome, acetazolamide-responsive | Neuromuscular junction diseases |
|  | MedGen | Potassium aggravated myotonia | Musculoskeletal diseases / Neurodegenerative diseases / Neuromuscular diseases |
|  | MedGen | Familial normokalemic periodic paralysis | Musculoskeletal diseases / Neuromuscular diseases / Metabolic diseases |
|  | MedGen | Paramyotonia congenita | Musculoskeletal diseases / Neuromuscular diseases |
|  | MedGen | Acetazolamide responsive myotonia | Musculoskeletal diseases / Neurodegenerative diseases / Neuromuscular diseases |
|  | MedGen | Potassium aggravated myotonia | Musculoskeletal diseases / Neurodegenerative diseases / Neuromuscular diseases |
|  | MedGen | Paramyotonia Congenita without Cold Paralysis | Musculoskeletal diseases / Neuromuscular diseases |
|  | MedGen | Severe Neonatal Episodic Laryngospasm | Respiration disorder |
| SCN4B | OMIM | Familial atrial fibrillation type 17 | Cardiac arrhythmias |
|  | OMIM | Long QT syndrome type 10 | Cardiac arrhythmias / Cardiac conduction system disease |
|  | Gene | Long QT syndrome type 10 | Cardiac arrhythmias / Cardiac conduction system disease |
|  | MedGen | Long QT syndrome type 10 | Cardiac arrhythmias / Cardiac conduction system disease |
|  | MedGen | Familial atrial fibrillation type 1 | Cardiac arrhythmias |
| SCN5A | OMIM | Familial atrial fibrillation type 10 | Cardiac arrhythmias |
|  | OMIM | Brugada Syndrome type 1 | Cardiac arrhythmias / Cardiac conduction system disease |
|  | OMIM | Cardiomyopathy dilated 1E | Cardiomyopathies |
|  | OMIM | heart block nonprogressive | Cardiac arrhythmias / Cardiac conduction system disease |
|  | OMIM | heart block progressive1A | Cardiac arrhythmias / Cardiac conduction system disease |
|  | OMIM | Long QT síndrome type 3 | Cardiac arrhythmias / Cardiac conduction system disease |
|  | OMIM | Sick sinus síndrome 1 | Cardiac arrhythmias / Cardiac conduction system disease |
|  | OMIM | Familial Ventricular Fibrillation type 1 | Cardiac arrhythmias |
|  | OMIM | sudden death syndrome | Pathological processes |
|  | Gene | Familial atrial fibrillation type 10 | Cardiac arrhythmias |
|  | Gene | Brugada Syndrome type 1 | Cardiac arrhythmias / Cardiac conduction system disease |
|  | Gene | Dilated cardiomyopathy 1E | Cardiomyopathies |
|  | Gene | Long QT síndrome type 3 | Cardiac arrhythmias / Cardiac conduction system disease |
|  | Gene | Paroxymal familial VF1 | Cardiac arrhythmias |
|  | Gene | Progressive familial heart block type 1A | Cardiac arrhythmias / Cardiac conduction system disease |
|  | Gene | Sick sinus syndrome 1, autosomal recessive | Cardiac arrhythmias / Cardiac conduction system disease |
|  | Gene | Sudden Infant Death Syndrome | Pathological processes |
|  | MedGen | Brugada Syndrome type 1 | Cardiac arrhythmias / Cardiac conduction system disease |
|  | MedGen | Paroxysimal Familial VF1 | Cardiac arrhythmias |
|  | MedGen | Long QT síndrome type 3 | Cardiac arrhythmias / Cardiac conduction system disease |
|  | MedGen | Dilated cardiomyopathy 1E | Cardiomyopathies |
|  | MedGen | Sudden Infant Death Syndrome | Pathological processes |
|  | MedGen | Progressive familial heart block type 1A | Cardiac arrhythmias / Cardiac conduction system disease |
|  | MedGen | Familial Atrial Fibrillation type 10 | Cardiac arrhythmias |
|  | MedGen | Familial Atrial Fibrillation type 1 | Cardiac arrhythmias |
|  | MedGen | Sick sinus syndrome 1, autosomal recessive | Cardiac arrhythmias / Cardiac conduction system disease |
| SCN9A | OMIM | Generalized epilepsy with febrile seizures plus type 7 | Febrile Seizures / Generalized Epilepsy |
|  | OMIM | erythermalgia primary | vascular diseases |
|  | OMIM | Familial Febrile seizures type 3B | febrile seizures |
|  | OMIM | Autosomal recessive congenital indifference to pain (HSAN2D) | peripheral nervous system diseases |
|  | OMIM | insensitivity to pain | peripheral nervous system diseases |
|  | OMIM | paroxysmal extreme pain disorder | peripheral nervous system diseases |
|  | OMIM | small fiber neuropathy | peripheral nervous system diseases |
|  | OMIM | modifier of Dravet syndrome | Generalized epilepsy |
|  | Gene | Generalized epilepsy with febrile seizures plus type 7 | Febrile seizures / generalized epilepsy |
|  | Gene | Hereditary sensory and autonomic neuropathy type IIA | Neurodegenerative diseases / Peripheral nervous system diseases |
|  | Gene | Indifference to pain, congenital, autosomal recessive | Peripheral nervous system diseases |
|  | Gene | Paroxysmal extreme pain disorder | Neurologic manifestations |
|  | Gene | Primary erythromelalgia | Vascular diseases |
|  | Gene | Severe myoclonic epilepsy of infancy | Generalized epilepsy |
|  | MedGen | Severe myoclonic epilepsy of infancy | Generalized epilepsy |
|  | MedGen | Generalized epilepsy with febrile seizures plus type 7 | Febrile seizures / generalized epilepsy |
|  | MedGen | Primary erythromelalgia | Vascular diseases |
|  | MedGen | Hereditary sensory and autonomic neuropathy type IIA | Neurodegenerative diseases / Peripheral nervous system diseases |
|  | MedGen | Indifference to pain, congenital, autosomal recessive | Peripheral nervous system diseases |
|  | MedGen | Paroxysmal extreme pain disorder | Neurologic manifestations |
|  | MedGen | Familial Febrile Seizures type 1 | Febrile seizures |
| KCNQ2 | OMIM | Early infantile epileptic encephalopathy type 7 | Infantile spasms (Generalized epilepsies / Epileptic syndromes) |
|  | OMIM | myokymia | neurologic manifestations |
|  | OMIM | Benign Neonatal Seizures type 1 | Benign neonatal epilepsy |
|  | Gene | Benign Familial Neonatal Seizures type 1 | Benign neonatal epilepsy |
|  | Gene | Early infantile epileptic encephalopathy type 7 | Infantile spasms (Generalized epilepsies / Epileptic syndromes) |
|  | MedGen | Benign Familial Neonatal Seizures type 1 | Benign neonatal epilepsy |
|  | MedGen | Early infantile epileptic encephalopathy type 7 | Infantile spasms (Generalized epilepsies / Epileptic syndromes) |
|  | MedGen | Early infantile epileptic encephalopathy type 1 | Infantile spasms (Generalized epilepsies / Epileptic syndromes) |
| KCNH2 | OMIM | Long QT Syndrome type 2 | Cardiac arrhythmias / Cardiac conduction system disease |
|  | OMIM | Short QT Syndrome type 1 | Cardiac arrhythmias / Cardiac conduction system disease |
|  | OMIM | susceptibility to LQTS2 | Cardiac arrhythmias / Cardiac conduction system disease |
|  | Gene | Long QT Syndrome type 2 | Cardiac arrhythmias / Cardiac conduction system disease |
|  | Gene | Short QT Syndrome type 1 | Cardiac arrhythmias / Cardiac conduction system disease |
|  | MedGen | Long QT Syndrome type 2 | Cardiac arrhythmias / Cardiac conduction system disease |
|  | MedGen | Short QT Syndrome type 1 | Cardiac arrhythmias / Cardiac conduction system disease |
| ANK3 | OMIM | Mental retardation, autosomal recessive 37 | Neurobehavioral manifestations |
|  | Gene | Mental retardation, autosomal recessive 37 | Neurobehavioral manifestations |
|  | MedGen | Mental retardation, autosomal recessive 37 | Neurobehavioral manifestations |
