# Supplementary material for: An integrative methodology based on protein-protein interaction networks for identification and functional annotation of disease-relevant genes applied to channelopathies
Source: BMC Bioinformatics. 2019 Nov 12;20:565. doi: 10.1186/s12859-019-3162-1 (PMC6849233; doi:10.1186/s12859-019-3162-1)
Supplement: Supplementary file 7 — Additional file 7. Dataset of genotype-phenotype relationships found through exhaustive review of the most relevant genes in channelopathies. Diseases linked to the nine relevant genes through the exhaustive review. Each phenotype is classified according to its MeSH category, as described in methods. Some evidences cannot be classified due to lack of information. [file 12859_2019_3162_MOESM7_ESM.docx]

**Table 7.1 Dataset of the genotype-phenotype relationships found through exhaustive review**. Diseases linked to the nine relevant genes through the exhaustive review. Each phenotype is classified according to its MeSH category. Some evidences cannot be classified due to lack of information.

| **Exhaustive review** | | | |
| --- | --- | --- | --- |
| **Gene** | **Disease** | **Lower-level category** | **References** |
| SCN1A | Severe myoclonic epilepsy of infancy or Dravet syndrome or EIEE6 | Myoclonic epilepsies (Generalized epilepsies / Epileptic syndromes) | [1–14] |
|  | Borderline, severe myoclonic epilepsy in infancy (Variants of DS [72]) | Generalized epilepsy | [2, 6, 7, 12] |
|  | intractable childhood epilepsy with generalized tonic-clonic seizures | Generalized epilepsy | [2, 4, 5, 7, 11–13] |
|  | infantile spasms or West syndrome | Generalized epilepsy | [2, 7, 12, 14] |
|  | Familial or sporadic hemiplegic migraine (type 3) | Headache disorders | [2, 7, 13, 15] |
|  | Generalized epilepsy with febrile seizures, plus (type 2) | Febrile seizures / generalized epilepsy | [2–4, 7, 12, 13, 16–18] |
|  | Lennox-Gastaut syndrome | Epileptic syndromes | [2, 13, 18] |
|  | migrating partial seizures of infancy | Partial Epilepsies | [2, 13, 18] |
|  | positive regulation of defense response to virus by host | Infections | [13] |
|  | Febrile seizure | Febrile seizures | [3, 12, 19] |
|  | Familial febrile seizure type 3 | Febrile seizures | [12, 16, 19] |
|  | Cryptogenic generalized epilepsy | Generalized epilepsy | [12] |
|  | Cryptogenic focal epilepsy | Partial epilepsies | [2, 11–13] |
|  | Myoclonic astatic Epilepsy | Myoclonic epilepsies (Generalized epilepsy / Epileptic syndromes) | [5, 12, 14, 20] |
|  | Severe idiopathic generalized epilepsy of infancy | Generalized epilepsy | [12, 20] |
|  | Rasmussen’s encephalitis | Encephalitis | [12, 21] |
|  | Vaccine encephalopathy | - | [10, 18] |
|  | Severe infantile multifocal epilepsy | Partial Epilepsies | [11, 18, 22] |
| SCN2A | Benign Familial Neonatal-Infantile Seizures, Benign Familial Infantile Seizures (Type 3) | Benign neonatal Epilepsy | [2–4, 13, 14, 18, 23–26] |
|  | Severe Myoclonic Epilepsy In Infancy | Generalized epilepsy | [4, 13, 14] |
|  | Intractable Childhood Epilepsy | Generalized epilepsy | [4, 14] |
|  | Generalized Epilepsy With Febrile Seizures Plus | Febrile seizures / generalized epilepsy | [2–4, 13, 14, 27] |
|  | Familial Febrile Seizures type 3B | Febrile seizures | [3, 28] |
|  | Dravet Syndrome | Myoclonic epilepsies (Generalized epilepsies / Epileptic syndromes) | [2, 13, 14, 18] |
|  | Early Infantile Epileptic Encephalopathy (Type 5) | Infantile spasms (Generalized epilepsies / Epileptic syndromes) | [13, 29, 30] |
|  | Early Infantile Epileptic Encephalopathy (Type 11) | Infantile spasms (Generalized epilepsies / Epileptic syndromes) | [13, 14, 31, 32] |
|  | Ohtahara syndrome | Infantile spasms (Generalized epilepsies / Epileptic syndromes) | [13, 29, 33, 34] |
|  | Infantile Spasms | Infantile spasms (Generalized epilepsies / Epileptic syndromes) | [4, 13, 32, 35] |
|  | Acute encephalitis with refractory repetitive partial seizures | Encephalitis / Partial epilepsies | [13] |
|  | Recurrent encephalopathy | - | [13, 31] |
|  | Episodic ataxia | Neurologic manifestations | [14, 24, 26] |
| SCN4A | Potassium-Aggravated Myotonia | Musculoskeletal diseases / Neurodegenerative diseases / Neuromuscular diseases | [13, 36–38] |
|  | Paramyotonia Congenita (OF VON EULENBURG | Musculoskeletal diseases / Neuromuscular diseases | [2, 13, 36–40] |
|  | Atypical Myotonia Congenita | Musculoskeletal diseases / Neurodegenerative diseases / Neuromuscular diseases | [2, 41] |
|  | Hyperkalaemic Periodic Paralysis (Type 1) or Hypokalaemic Periodic Paralysis (Type 1,2) | Musculoskeletal diseases / Neuromuscular diseases / Metabolic diseases | [2, 13, 36–38, 42, 43] |
|  | Acetazolamide-Responsive Myotonia | Musculoskeletal diseases / Neurodegenerative diseases / Neuromuscular diseases | [2, 37, 44, 45] |
|  | Myasthenic Syndrome, Congenital, 16 | Neuromuscular junction diseases | [37, 44, 46, 47] |
|  | Brugada syndrome | Cardiac arrythmias | [48] |
| SCN4B | Familial atrial fibrillation (type 17) | Cardiac arrhythmias | [49–52] |
|  | Long QT Syndrome (Type 3,10) | Cardiac arrhythmias / Cardiac conduction system disease | [49, 53, 54] |
|  | Brugada syndrome | Cardiac arrhythmias / Cardiac conduction system disease | [49, 55] |
|  | progressive cardiac conduction defect | Cardiac arrhythmias / Cardiac conduction system disease | [49] |
|  | sick sinus node syndrome | Cardiac arrhythmias / Cardiac conduction system disease | [49] |
|  | dilated cardiomyopathy | Cardiomyopathies | [49] |
| SCN5A | Atrial Fibrillation, Familial, 10 | Cardiac arrhythmias | [49, 51, 52, 56–59] |
|  | Atrial Standstill 1, Digenic | Cardiac arrhythmias / Cardiac conduction system disease / Cardiomyopathies | [58, 59] |
|  | Sudden Cardiac Death | Heart arrest / Pathological Processes | [49, 60, 61] |
|  | Brugada Syndrome type 1 | Cardiac arrhythmias / Cardiac conduction system disease | [48, 49, 51, 60–63] |
|  | Idiopathic Ventricular Fibrillation, Not Brugada Type | Cardiac arrhythmias | [61, 64–66] |
|  | Cardiomyopathy, Familial Dilated (type 1E) | Cardiomyopathies | [49, 58–60] |
|  | Familial Progressive Cardiac Conduction Defect | Cardiac arrhythmias / Cardiac conduction system disease | [49, 51, 67, 68] |
|  | Long QT Syndrome 3 or Romano-Ward Syndrome | Cardiac arrhythmias / Cardiac conduction system disease | [2, 49, 51, 55, 58, 60, 61, 68–74] |
|  | Sudden Infant Death Syndrome | Heart arrest / Pathological Processes | [49, 59–61, 68, 75–77] |
|  | Sick Sinus Syndrome 1, Autosomal Recessive | Cardiac arrhythmias / Cardiac conduction system disease | [49, 58, 59, 78, 79] |
|  | Ventricular Fibrillation, Familial, (type 1) | Cardiac arrhythmias | [66] |
| SCN9A | Channelopathy-associated congenital insensitivity to pain | Peripheral Nervous system diseases | [80–82] |
|  | Dravet Syndrome (Modifier Of) Or EIEE6 | Myoclonic epilepsies (Generalized epilepsies / Epileptic syndromes) | [3, 6, 12, 18] |
|  | SMEB or Intractable Childhood Epilepsy | Generalized epilepsy | [3] |
|  | Erythermalgia, Primary | Vascular diseases | [83–87] |
|  | Generalized Epilepsy With Febrile Seizures Plus (Type 7) | Febrile seizures / generalized epilepsy | [3, 19, 28, 88] |
|  | Hereditary sensory and autonomic neuropathy type 2 | Neurodegenerative diseases / Peripheral nervous system diseases | [13, 89] |
|  | Idiopathic small fiber neuropathy | Peripheral nervous system diseases | [13] |
|  | Painful peripheral neuropathy | Peripheral nervous system diseases | [13, 90, 91] |
|  | Paroxysmal Extreme Pain Disorder | Neurologic manifestations | [13, 84, 92, 93] |
|  | congenital insensitivity to pain | Peripheral nervous system diseases | [13, 92] |
|  | Familial febrile seizures | Febrile seizures | [3, 18, 28] |
| KCNQ2 | Benign familial neonatal epilepsy (or convulsions) | Benign neonatal epilepsy | [94–98] |
|  | Benign familial neonatal convulsions with myokymia | Benign neonatal epilepsy / neurologic manifestations | [99] |
|  | Neonatal epilepsy | Benign neonatal epilepsy | [25, 100] |
|  | myokymia (type 2) | neurologic manifestations | [13, 91] |
|  | Peripheral nerve hyperexcitability | Peripheral nervous system diseases | [13, 91] |
|  | Early infantile epileptic encephalopathy (type 7) and Severe epileptic encephalopathies | Infantile spasms (Generalized epilepsies / Epileptic syndromes) | [13, 25, 91, 96, 100] |
| KCNH2 | Long QT syndrome (type 2) | Cardiac arrhythmias / Cardiac conduction system disease | [69, 70, 72, 73, 101–105] |
|  | Short QT syndrome (type 1) | Cardiac arrhythmias / Cardiac conduction system disease | [106–109] |
|  | Infantile subit death syndrome | Heart arrest / Pathological Processes | [74, 77, 105, 108, 110] |
|  | Brugada syndrome | Cardiac arrhythmias / Cardiac conduction system disease | [57, 71, 108] |
|  | Cardiovascular disease (arrhythmias) | Cardiac arrhythmias | [105, 107] |
|  | Familial Atrial fibrillation | Cardiac arrhythmias | [57, 108, 111] |
| ANK3 | Autosomic recessive mental disability 37 | Neurobehavioral manifestations | [112] |
|  | Autism Spectrum disorder | Mental disorders | [113, 114] |
|  | Early-onset Alzheimer disease | Brain diseases / Neurodegenerative diseases / Mental disorders | [115] |
|  | Schizophrenia | Mental disorders | [112, 116–118] |
|  | Bipolar disorder | Mental disorders | [112, 117, 119–125] |

a1. Marini C, Scheffer IE, Nabbout R, Suls A, De Jonghe P, Zara F, et al. The genetics of Dravet syndrome. Epilepsia. 2011;52 SUPPL. 2:24–9. doi:10.1111/j.1528-1167.2011.02997.x.

2. Mulley JC, Scheffer IE, Petrou S, Dibbens LM, Berkovic SF, Harkin LA. SCN1A mutations and epilepsy. Hum Mutat. 2005;25:535–42. doi:10.1002/humu.20178.

3. Singh NA, Pappas C, Dahle EJ, Claes LRF, Pruess TH, De Jonghe P, et al. A role of SCN9A in human epilepsies, as a cause of febrile seizures and as a potential modifier of Dravet syndrome. PLoS Genet. 2009;5:e1000649. doi:10.1371/journal.pgen.1000649.

4. Ogiwara I, Ito K, Sawaishi Y, Osaka H, Mazaki E, Inoue I, et al. De novo mutations of voltage-gated sodium channel alphaII gene SCN2A in intractable epilepsies. Neurology. 2009;73:1046–53. doi:10.1212/WNL.0b013e3181b9cebc.

5. Griffin A, Hamling KR, Knupp K, Hong S, Lee LP, Baraban SC. Clemizole and modulators of serotonin signalling suppress seizures in Dravet syndrome. Brain. 2017;140:aww342. doi:10.1093/brain/aww342.

6. Steel D, Symonds JD, Zuberi SM, Brunklaus A. Dravet syndrome and its mimics: Beyond *SCN1A*. Epilepsia. 2017;58:1807–16. doi:10.1111/epi.13889.

7. Fujiwara T, Sugawara T, Mazaki-Miyazaki E, Takahashi Y, Fukushima K, Watanabe M, et al. Mutations of sodium channel alpha subunit type 1 (SCN1A) in intractable childhood epilepsies with frequent generalized tonic-clonic seizures. Brain. 2003;126:531–46.

8. Nabbout R, Gennaro E, Dalla Bernardina B, Dulac O, Madia F, Bertini E, et al. Spectrum of SCN1A mutations in severe myoclonic epilepsy of infancy. Neurology. 2003;60:1961–7. doi:10.1212/01.WNL.0000069463.41870.2F.

9. Sugawara T, Mazaki-Miyazaki E, Fukushima K, Shimomura J, Fujiwara T, Hamano S, et al. Frequent mutations of SCN1A in severe myoclonic epilepsy in infancy. Neurology. 2002;58:1122–4. doi:10.1212/WNL.58.7.1122.

10. Claes L, Del-Favero J, Ceulemans B, Lagae L, Broeckhoven C Van, De Jonghe P. De Novo Mutations in the Sodium-Channel Gene SCN1A Cause Severe Myoclonic Epilepsy of Infancy. Am J Hum Genet. 2001;68:1327–32.

11. Harkin LA, McMahon JM, Iona X, Dibbens L, Pelekanos JT, Zuberi SM, et al. The spectrum of SCN1A-related infantile epileptic encephalopathies. Brain. 2007;130:843–52. doi:10.1093/brain/awm002.

12. Parihar R, Ganesh S. The SCN1A gene variants and epileptic encephalopathies. J Hum Genet. 2013;58:573–80. doi:10.1038/jhg.2013.77.

13. Spillane J, Kullmann DM, Hanna MG. Genetic neurological channelopathies: molecular genetics and clinical phenotypes. J Neurol Neurosurg Psychiatry. 2016;87:37–48.

14. Shi X, Yasumoto S, Kurahashi H, Nakagawa E, Fukasawa T, Uchiya S, et al. Clinical spectrum of SCN2A mutations. Brain Dev. 2012;34:541–5. doi:10.1016/j.braindev.2011.09.016.

15. Dichgans M, Freilinger T, Eckstein G, Babini E, Lorenz-Depiereux B, Biskup S, et al. Mutation in the neuronal voltage-gated sodium channel SCN1A in familial hemiplegic migraine. Lancet. 2005;366:371–7. doi:10.1016/S0140-6736(05)66786-4.

16. Alekov AK, Rahman MM, Mitrovic N, Lehmannhorn F, Lerche H. A sodium channel mutation causing epilepsy in man exhibits subtle defects in fast inactivation and activation in vitro. J Physiol. 2000;529:533–9. doi:10.1111/j.1469-7793.2000.00533.x.

17. Kristian H, Amstel P Van. Mutations of SCN1A, encoding a neuronal sodium channel, in two families with GEFS+2. Nat Genet. 2000;24:316–20.

18. Oliva M, Berkovic SF, Petrou S. Sodium channels and the neurobiology of epilepsy. Epilepsia. 2012;53:1849–59. doi:10.1111/j.1528-1167.2012.03631.x.

19. Mantegazza M, Gambardella A, Rusconi R, Schiavon E, Annesi F, Cassulini RR, et al. Identification of an Nav1.1 sodium channel (SCN1A) loss-of-function mutation associated with familial simple febrile seizures. Proc Natl Acad Sci U S A. 2005;102:18177–82. doi:10.1073/pnas.0506818102.

20. Ebach K, Joos H, Doose H, Stephani U, Kurlemann G, Fiedler B, et al. SCN1A Mutation Analysis in Myoclonic Astatic Epilepsy and Severe Idiopathic Generalized Epilepsy of Infancy with Generalized Tonic-Clonic Seizures. Neuropediatrics. 2005;36:210–3. doi:10.1055/s-2005-865607.

21. Ohmori I, Ouchida M, Kobayashi K, Jitsumori Y, Inoue T, Shimizu K, et al. Rasmussen encephalitis associated with SCN1Amutation. Epilepsia. 2008;49:521–6. doi:10.1111/j.1528-1167.2007.01411.x.

22. Carranza Rojo D, Hamiwka L, McMahon JM, Dibbens LM, Arsov T, Suls A, et al. De novo SCN1A mutations in migrating partial seizures of infancy. Neurology. 2011;77:380–3. doi:10.1212/WNL.0b013e318227046d.

23. Heron SE, Crossland KM, Andermann E, Phillips HA, Hall AJ, Bleasel A, et al. Sodium-channel defects in benign familial neonatal-infantile seizures. Lancet (London, England). 2002;360:851–2. doi:10.1016/S0140-6736(02)09968-3.

24. Leach EL, van Karnebeek CDM, Townsend KN, Tarailo-Graovac M, Hukin J, Gibson WT. Episodic ataxia associated with a de novo SCN2A mutation. Eur J Paediatr Neurol. 2016;20:772–6. doi:10.1016/j.ejpn.2016.05.020.

25. Zeng Q, Yang X, Zhang J, Liu A, Yang Z, Liu X, et al. Genetic analysis of benign familial epilepsies in the first year of life in a Chinese cohort. J Hum Genet. 2018;63:9–18. doi:10.1038/s10038-017-0359-x.

26. Gorman KM, King MD. SCN2A p.Ala263Val Variant a Phenotype of Neonatal Seizures Followed by Paroxysmal Ataxia in Toddlers. Pediatr Neurol. 2017;67:111–2. doi:10.1016/j.pediatrneurol.2016.11.008.

27. Meisler MH, O’Brien JE, Sharkey LM. Sodium channel gene family: epilepsy mutations, gene interactions and modifier effects. J Physiol. 2010;588:1841–8. doi:10.1113/jphysiol.2010.188482.

28. Moulard B, Chaigne D, Malafosse A. Clinical heterogeneity in pedigrees with 2q-linked febrile seizures. Ann Neurol. 2000;47:839–40. http://www.ncbi.nlm.nih.gov/pubmed/10852559.

29. Nakamura K, Kato M, Osaka H, Yamashita S, Nakagawa E, Haginoya K, et al. Clinical spectrum of SCN2A mutations expanding to Ohtahara syndrome. 2013. doi:10.1212/WNL.0b013e3182a43e57.

30. Ko A, Youn SE, Kim SH, Lee JS, Kim S, Choi JR, et al. Targeted gene panel and genotype-phenotype correlation in children with developmental and epileptic encephalopathy. Epilepsy Res. 2018;141:48–55. doi:10.1016/j.eplepsyres.2018.02.003.

31. Fukasawa T, Kubota T, Negoro T, Saitoh M, Mizuguchi M, Ihara Y, et al. A case of recurrent encephalopathy with SCN2A missense mutation. Brain Dev. 2015;37:631–4. doi:10.1016/j.braindev.2014.10.001.

32. Dilena R, Striano P, Gennaro E, Bassi L, Olivotto S, Tadini L, et al. Efficacy of sodium channel blockers in SCN2A early infantile epileptic encephalopathy. Brain Dev. 2017;39:345–8. doi:10.1016/j.braindev.2016.10.015.

33. Su D-J, Lu J-F, Lin L-J, Liang J-S, Hung K-L. SCN2A mutation in an infant presenting with migrating focal seizures and infantile spasm responsive to a ketogenic diet. Brain Dev. 2018. doi:10.1016/j.braindev.2018.03.005.

34. Wolff M, Johannesen KM, Hedrich UBS, Masnada S, Rubboli G, Gardella E, et al. Genetic and phenotypic heterogeneity suggest therapeutic implications in SCN2A-related disorders. Brain. 2017;140:1316–36. doi:10.1093/brain/awx054.

35. Ben-Shalom R, Keeshen CM, Berrios KN, An JY, Sanders SJ, Bender KJ. Opposing Effects on Na V 1.2 Function Underlie Differences Between SCN2A Variants Observed in Individuals With Autism Spectrum Disorder or Infantile Seizures. Biol Psychiatry. 2017;82:224–32. doi:10.1016/j.biopsych.2017.01.009.

36. Corrochano S, Männikkö R, Joyce PI, McGoldrick P, Wettstein J, Lassi G, et al. Novel mutations in human and mouse SCN4A implicate AMPK in myotonia and periodic paralysis. Brain. 2014;137:3171–85. doi:10.1093/brain/awu292.

37. Nicole S, Fontaine B. Skeletal muscle sodium channelopathies. Curr Opin Neurol. 2015;28:508–514xs. doi:10.1097/WCO.0000000000000238.

38. Abicht A, Müller J, Lochmüller H. Congenital Myasthenic Syndromes. University of Washington, Seattle; 1993. http://www.ncbi.nlm.nih.gov/pubmed/20301347. Accessed 5 Mar 2018.

39. Stunnenberg BC, Raaphorst J, Deenen JCW, Links TP, Wilde AA, Verbove DJ, et al. Prevalence and mutation spectrum of skeletal muscle channelopathies in the Netherlands. Neuromuscul Disord. 2018;28:402–7. doi:10.1016/j.nmd.2018.03.006.

40. Palmio J, Sandell S, Hanna MG, Männikkö R, Penttilä S, Udd B. Predominantly myalgic phenotype caused by the c.3466G&gt;A p.A1156T mutation in *SCN4A* gene. Neurology. 2017;88:1520–7. doi:10.1212/WNL.0000000000003846.

41. Yamamoto J, Hokkoku K, Hatanaka Y, Sakoda S, Yuan J-H, Sonoo M. An unusual case of sodium channel myotonia with transient weakness upon initiating movements which is characteristic in Becker disease. Rinsho Shinkeigaku. 2017;57:287–92. doi:10.5692/clinicalneurol.cn-000980.

42. Allard B, Fuster C. When muscle Ca ^2+^ channels carry monovalent cations through gating pores: insights into the pathophysiology of type 1 hypokalaemic periodic paralysis. J Physiol. 2018;596:2019–27. doi:10.1113/JP274955.

43. Hong D, Luan X, Chen B, Zheng R, Zhang W, Wang Z, et al. Both hypokalaemic and normokalaemic periodic paralysis in different members of a single family with novel R1129Q mutation in SCN4A gene. J Neurol Neurosurg Psychiatry. 2010;81:703–4. doi:10.1136/jnnp.2009.177451.

44. Catterall WA. International Union of Pharmacology. XLVII. Nomenclature and Structure-Function Relationships of Voltage-Gated Sodium Channels. Pharmacol Rev. 2005;57:397–409. doi:10.1124/pr.57.4.4.

45. Moreira SD, Barreto R, Roriz JM. Becker myotonia-a recently identified mutation in iberian descendants with apparent acetazolamide-responsive phenotype. Muscle Nerve. 2015;51:933–4. doi:10.1002/mus.24534.

46. Engel AG. Genetic basis and phenotypic features of congenital myasthenic syndromes. In: Handbook of clinical neurology. 2018. p. 565–89. doi:10.1016/B978-0-444-64076-5.00037-5.

47. Männikkö R, Wong L, Tester DJ, Thor MG, Sud R, Kullmann DM, et al. Dysfunction of NaV1.4, a skeletal muscle voltage-gated sodium channel, in sudden infant death syndrome: a case-control study. Lancet. 2018;391:1483–92. doi:10.1016/S0140-6736(18)30021-7.

48. Bissay V, Van Malderen SCH, Keymolen K, Lissens W, Peeters U, Daneels D, et al. SCN4A variants and Brugada syndrome: phenotypic and genotypic overlap between cardiac and skeletal muscle sodium channelopathies. Eur J Hum Genet. 2016;24:400–7. doi:10.1038/ejhg.2015.125.

49. Zeng Z, Zhou J, Hou Y, Liang X, Zhang Z, Xu X, et al. Electrophysiological Characteristics of a SCN5A Voltage Sensors Mutation R1629Q Associated With Brugada Syndrome. PLoS One. 2013;8:e78382. doi:10.1371/journal.pone.0078382.

50. Fang W-Y. Mutations of the SCN4B-encoded sodium channel $β$4 subunit in familial atrial fibrillation. Int J Mol Med. 2013. doi:10.3892/ijmm.2013.1355.

51. Savio-Galimberti E, Darbar D. Atrial Fibrillation and SCN5A Variants. Card Electrophysiol Clin. 2014;6:741–8. doi:10.1016/j.ccep.2014.07.006.

52. Husser D, Ueberham L, Hindricks G, Büttner P, Ingram C, Weeke P, et al. Rare variants in genes encoding the cardiac sodium channel and associated compounds and their impact on outcome of catheter ablation of atrial fibrillation. PLoS One. 2017;12:e0183690. doi:10.1371/journal.pone.0183690.

53. Riuró H, Campuzano O, Arbelo E, Iglesias A, Batlle M, Pérez-Villa F, et al. A missense mutation in the sodium channel β1b subunit reveals SCN1B as a susceptibility gene underlying long QT syndrome. Hear Rhythm. 2014;11:1202–9. doi:10.1016/j.hrthm.2014.03.044.

54. Medeiros-Domingo A, Kaku T, Tester DJ, Iturralde-Torres P, Itty A, Ye B, et al. SCN4B -Encoded Sodium Channel β4 Subunit in Congenital Long-QT Syndrome. Circulation. 2007;116:134–42. doi:10.1161/CIRCULATIONAHA.106.659086.

55. Watanabe H, Koopmann TT, Le Scouarnec S, Yang T, Ingram CR, Schott J-J, et al. Sodium channel $β$1 subunit mutations associated with Brugada syndrome and cardiac conduction disease in humans. J Clin Invest. 2008;118:2260–8. doi:10.1172/JCI33891.

56. Conte G, Caputo ML, Volders PGA, Luca A, Mainardi L, Schotten U, et al. Concealed abnormal atrial phenotype in patients with Brugada syndrome and no history of atrial fibrillation. Int J Cardiol. 2018;253:66–70. doi:10.1016/j.ijcard.2017.09.214.

57. Johnson JN, Tester DJ, Perry J, Salisbury BA, Reed CR, Ackerman MJ. Prevalence of early-onset atrial fibrillation in congenital long QT syndrome. Hear Rhythm. 2008;5:704–9. doi:10.1016/j.hrthm.2008.02.007.

58. Remme CA. Cardiac sodium channelopathy associated with SCN5A mutations: electrophysiological, molecular and genetic aspects. J Physiol. 2013;591:4099–116. doi:10.1113/jphysiol.2013.256461.

59. Savio-Galimberti E, Gollob MH, Darbar D. Voltage-Gated Sodium Channels: Biophysics, Pharmacology, and Related Channelopathies. Front Pharmacol. 2012;3 July:1–19. doi:10.3389/fphar.2012.00124.

60. Kinoshita K, Takahashi H, Hata Y, Nishide K, Kato M, Fujita H, et al. SCN5A(K817E), a novel Brugada syndrome–associated mutation that alters the activation gating of NaV1.5 channel. Hear Rhythm. 2016;13:1113–20. doi:10.1016/j.hrthm.2016.01.008.

61. Chagot B, Potet F, Balser JR, Chazin WJ. Solution NMR Structure of the C-terminal EF-hand Domain of Human Cardiac Sodium Channel Na V 1.5. J Biol Chem. 2009;284:6436–45. doi:10.1074/jbc.M807747200.

62. Michowitz Y, Milman A, Sarquella-Brugada G, Andorin A, Champagne J, Postema PG, et al. Fever-related arrhythmic events in the multicenter Survey on Arrhythmic Events in Brugada Syndrome. Hear Rhythm. 2018. doi:10.1016/j.hrthm.2018.04.007.

63. Matsumura H, Nakano Y, Ochi H, Onohara Y, Sairaku A, Tokuyama T, et al. H558R, a common SCN5A polymorphism, modifies the clinical phenotype of Brugada syndrome by modulating DNA methylation of SCN5A promoters. J Biomed Sci. 2017;24:91. doi:10.1186/s12929-017-0397-x.

64. Ding D-B, Fan L-L, Xiao Z, Huang H, Chen Y-Q, Guo S, et al. A novel mutation of dipeptidyl aminopeptidase-like protein-6 in a family with suspicious idiopathic ventricular fibrillation. QJM An Int J Med. 2018;111:373–7. doi:10.1093/qjmed/hcy033.

65. Akai J, Makita N, Sakurada H, Shirai N, Ueda K, Kitabatake A, et al. A novel SCN5A mutation associated with idiopathic ventricular fibrillation without typical ECG findings of Brugada syndrome. FEBS Lett. 2000;479:29–34. http://www.ncbi.nlm.nih.gov/pubmed/10940383.

66. Boehringer T, Bugert P, Borggrefe M EE. SCN5A mutations and polymorphisms in patients with ventricular fibrillation during acute myocardial infarction. Mol Med Rep. 2014;10:2039–44. doi:10.3892/mmr.2014.2401.

67. Baruteau A-E, Pass RH, Thambo J-B, Behaghel A, Le Pennec S, Perdreau E, et al. Congenital and childhood atrioventricular blocks: pathophysiology and contemporary management. Eur J Pediatr. 2016;175:1235–48. doi:10.1007/s00431-016-2748-0.

68. Wu L, Nishiyama K, Hollyfield JG, Wang Q. Localization of Nav1.5 sodium channel protein in the mouse brain. Neuroreport. 2002;13:2547–51. doi:10.1097/01.wnr.0000052322.62862.a5.

69. Crotti L, Lundquist AL, Insolia R, Pedrazzini M, Ferrandi C, De Ferrari GM, et al. KCNH2-K897T is a genetic modifier of latent congenital long-QT syndrome. Circulation. 2005;112:1251–8. doi:10.1161/CIRCULATIONAHA.105.549071.

70. Ito S, Taketani T, Sugamori T, Okada T, Sato H, Adachi T, et al. A case of long QT syndrome having compound mutations of KCNH2 and SCN5A. J Cardiol Cases. 2012;6:e170--e172. doi:10.1016/j.jccase.2012.07.004.

71. Wang QI, Ohno S, Ding W-GG, Fukuyama M, Miyamoto A, Itoh H, et al. Gain-of-function KCNH2 mutations in patients with Brugada syndrome. J Cardiovasc Electrophysiol. 2014;25:522–30. doi:10.1111/jce.12361.

72. Chae H, Kim J, Lee GD, Jang W, Park J, Jekarl DW, et al. Considerations when using next-generation sequencing for genetic diagnosis of long-QT syndrome in the clinical testing laboratory. Clin Chim Acta. 2017;464:128–35. doi:10.1016/j.cca.2016.11.013.

73. Al-Hassnan ZN, Al-Fayyadh M, Al-Ghamdi B, Shafquat A, Mallawi Y, Al-Hadeq F, et al. Clinical profile and mutation spectrum of long QT syndrome in Saudi Arabia: The impact of consanguinity. Hear Rhythm. 2017;14:1191–9. doi:10.1016/j.hrthm.2017.04.028.

74. Brugada R, Hong K, Dumaine R, Cordeiro J, Gaita F, Borggrefe M, et al. Sudden death associated with short-QT syndrome linked to mutations in HERG. Circulation. 2004;109:30–5. doi:10.1161/01.CIR.0000109482.92774.3A.

75. Denti F, Bentzen BH, Wojciak J, Thomsen NM, Scheinman M, Schmitt N. Multiple genetic variations in sodium channel subunits in a case of sudden infant death syndrome. PACE - Pacing Clin Electrophysiol. 2018;41:620–6. doi:10.1111/pace.13328.

76. Ackerman MJ, Siu BL, Sturner WQ, Tester DJ, Valdivia CR, Makielski JC, et al. Postmortem molecular analysis of SCN5A defects in sudden infant death syndrome. JAMA. 2001;286:2264–9. doi:10.1001/jama.286.18.2264.

77. Otagiri T, Kijima K, Osawa M, Ishii K, Makita N, Matoba R, et al. Cardiac Ion Channel Gene Mutations in Sudden Infant Death Syndrome. Pediatr Res. 2008;64:482–7. doi:10.1203/PDR.0b013e3181841eca.

78. Lee YS, Olaopa MA, Jung BC, Lee SH, Shin DG, Park HS, et al. Genetic Variation of *SCN5A* in Korean Patients with Sick Sinus Syndrome. Korean Circ J. 2016;46:63. doi:10.4070/kcj.2016.46.1.63.

79. Butters TD, Aslanidi O V, Inada S, Boyett MR, Hancox JC, Lei M, et al. Mechanistic Links Between Na + Channel (SCN5A) Mutations and Impaired Cardiac Pacemaking in Sick Sinus Syndrome. Circ Res. 2010;107:126–37. doi:10.1161/CIRCRESAHA.110.219949.

80. Rajasekharan S, Martens L, Domingues L, Cauwels R. SCN9A channelopathy associated autosomal recessive Congenital Indifference to Pain. A case report. Eur J Paediatr Dent. 2017;18:66–8. doi:10.23804/ejpd.2017.18.01.14.

81. Cox JJ, Reimann F, Nicholas AK, Thornton G, Roberts E, Springell K, et al. An SCN9A channelopathy causes congenital inability to experience pain. Nature. 2006;444:894–8. doi:10.1038/nature05413.

82. Goldberg YP, MacFarlane J, MacDonald ML, Thompson J, Dube M-P, Mattice M, et al. Loss-of-function mutations in the Nav1.7 gene underlie congenital indifference to pain in multiple human populations. Clin Genet. 2007;71:311–9. doi:10.1111/j.1399-0004.2007.00790.x.

83. Klein-Weigel PF, Volz TS, Richter JG. Erythromelalgia. Vasa. 2018;47:91–7. doi:10.1024/0301-1526/a000675.

84. Catterall WA, Yu FH. Painful Channels. Neuron. 2006;52:743–4. doi:10.1016/j.neuron.2006.11.017.

85. Hisama FM, Dib-Hajj SD, Waxman SG. SCN9A-Related Inherited Erythromelalgia. Seattle (WA): University of Washington, Seattle. https://www.ncbi.nlm.nih.gov/books/NBK1163/.

86. Michiels JJ, te Morsche RHM, Jansen JBMJ, Drenth JPH. Autosomal Dominant Erythermalgia Associated With a Novel Mutation in the Voltage-Gated Sodium Channel a Subunit Nav1.7. Arch Neurol. 2005;62:1587–90. doi:10.1001/archneur.62.10.1587.

87. Yang Y, Wang Y, Li S, Xu Z, Li H, Ma L, et al. Mutations in SCN9A, encoding a sodium channel alpha subunit, in patients with primary erythermalgia. J Med Genet. 2004;41:171–4. http://www.ncbi.nlm.nih.gov/pubmed/14985375.

88. Cen Z, Lou Y, Guo Y, Wang J, Feng J. Q10R mutation in SCN9A gene is associated with generalized epilepsy with febrile seizures plus. Seizure. 2017;50:186–8. doi:10.1016/j.seizure.2017.06.023.

89. Faber CG, Hoeijmakers JGJ, Ahn H-S, Cheng X, Han C, Choi J-S, et al. Gain of function NaV1.7 mutations in idiopathic small fiber neuropathy. Ann Neurol. 2012;71:26–39. doi:10.1002/ana.22485.

90. Wadhawan S, Pant S, Golhar R, Kirov S, Thompson J, Jacobsen L, et al. Na _V_ channel variants in patients with painful and nonpainful peripheral neuropathy. Neurol Genet. 2017;3:e207. doi:10.1212/NXG.0000000000000207.

91. Milh M, Lacoste C, Cacciagli P, Abidi A, Sutera-Sardo J, Tzelepis I, et al. Variable clinical expression in patients with mosaicism for KCNQ2 mutations. Am J Med Genet Part A. 2015;167:2314–8. doi:10.1002/ajmg.a.37152.

92. Fouillet A, Watson JF, Piekarz AD, Huang X, Li B, Priest B, et al. Characterisation of Nav1.7 functional expression in rat dorsal root ganglia neurons by using an electrical field stimulation assay. Mol Pain. 2017;13:1744806917745179. doi:10.1177/1744806917745179.

93. Fertleman CR, Baker MD, Parker KA, Moffatt S, Elmslie F V, Abrahamsen B, et al. SCN9A Mutations in Paroxysmal Extreme Pain Disorder: Allelic Variants Underlie Distinct Channel Defects and Phenotypes. Neuron. 2006;52:767–74. doi:10.1016/j.neuron.2006.10.006.

94. Ambrosino P, Freri E, Castellotti B, Soldovieri MV, Mosca I, Manocchio L, et al. Kv7.3 Compound Heterozygous Variants in Early Onset Encephalopathy Reveal Additive Contribution of C-Terminal Residues to PIP2-Dependent K+ Channel Gating. Mol Neurobiol. 2018;55:7009–24. doi:10.1007/s12035-018-0883-5.

95. Castaldo P, del Giudice EM, Coppola G, Pascotto A, Annunziato L, Taglialatela M. Benign familial neonatal convulsions caused by altered gating of KCNQ2/KCNQ3 potassium channels. J Neurosci. 2002;22:RC199. doi:20025989.

96. Miceli F, Soldovieri MV, Joshi N, Weckhuysen S, Cooper E, Taglialatela M. KCNQ2-Related Disorders. University of Washington, Seattle; 1993. doi:NBK32534.

97. Nanda A. Singh, Carole Charlier, Dora Stauffer, Barbara R. DuPont, Robin J. Leach, Roberta Melis, Gabriel M. Ronen, Ingrid Bjerre, Thomas Quattlebaum, Jerome V. Murphy, Malcolm L. McHarg8, David Gagnon9, Teodoro O. Rosales, Andy Peiffer VEA& ML. A novel potassium channel gene, KCNQ2, is mutated in an inherited epilepsy of newborns. Nat Genet. 1998;18:25–9.

98. Steinlein OK, Stoodt J, Biervert C, Janz D, Sander T. The voltage gated potassium channel KCNQ2 and idiopathic generalized epilepsy. Neuroreport. 1999;10:1163–6. http://www.ncbi.nlm.nih.gov/pubmed/10363917.

99. Gutman GA. International Union of Pharmacology. LIII. Nomenclature and Molecular Relationships of Voltage-Gated Potassium Channels. Pharmacol Rev. 2005;57:473–508. doi:10.1124/pr.57.4.10.

100. Abidi A, Devaux JJ, Molinari F, Alcaraz G, Michon F-X, Sutera-Sardo J, et al. A recurrent KCNQ2 pore mutation causing early onset epileptic encephalopathy has a moderate effect on M current but alters subcellular localization of Kv7 channels. Neurobiol Dis. 2015;80:80–92. doi:10.1016/j.nbd.2015.04.017.

101. Fujii Y, Matsumoto Y, Hayashi K, Ding W-G, Tomita Y, Fukumoto D, et al. Contribution of a KCNH2 variant in genotyped long QT syndrome: Romano–Ward syndrome under double mutations and acquired long QT syndrome under heterozygote. J Cardiol. 2017;70:74–9. doi:10.1016/j.jjcc.2016.09.010.

102. Gianulis EC, Trudeau MC. Rescue of Aberrant Gating by a Genetically Encoded PAS (Per-Arnt-Sim) Domain in Several Long QT Syndrome Mutant Human Ether-á-go-go -related Gene Potassium Channels. J Biol Chem. 2011;286:22160–9. doi:10.1074/jbc.M110.205948.

103. Johnson JN, Hofman N, Haglund CM, Cascino GD, Wilde AAM, Ackerman MJ. Identification of a possible pathogenic link between congenital long QT syndrome and epilepsy. Neurology. 2009;72:224–31. doi:10.1212/01.wnl.0000335760.02995.ca.

104. Keller Md DI, Grenier Msc J, Phd GC, Dubouloz Phd F, Osswald Md S, Brink Phd M, et al. Characterization of novel KCNH2 mutations in type 2 long QT syndrome manifesting as seizures. Can J Cardiol. 2009;25:455–62. http://www.pubmedcentral.nih.gov/articlerender.fcgi?artid=PMC2732373.

105. Sanguinetti MC. HERG1 channelopathies. Pflugers Arch. 2010;460:265–76. doi:10.1007/s00424-009-0758-8.

106. El‐Battrawy I, Lan H, Cyganek L, Zhao Z, Li X, Buljubasic F, et al. Modeling Short QT Syndrome Using Human‐Induced Pluripotent Stem Cell–Derived Cardiomyocytes. J Am Heart Assoc. 2018;7:e007394. doi:10.1161/JAHA.117.007394.

107. Cordeiro JM, Brugada R, Wu YS, Hong K, Dumaine R, Gussak I, et al. Modulation of I(Kr) inactivation by mutation N588K in KCNH2: a link to arrhythmogenesis in short QT syndrome. Cardiovasc Res. 2005;67:498–509. doi:10.1016/j.cardiores.2005.02.018.

108. Hong K, Bjerregaard P, Gussak I, Brugada R. Short QT syndrome and atrial fibrillation caused by mutation in KCNH2. J Cardiovasc Electrophysiol. 2005;16:394–6. doi:10.1046/j.1540-8167.2005.40621.x.

109. Sun Y, Quan X-Q, Fromme S, Cox RH, Zhang P, Zhang L, et al. A novel mutation in the KCNH2 gene associated with short QT syndrome. J Mol Cell Cardiol. 2011;50:433–41. doi:10.1016/j.yjmcc.2010.11.017.

110. Christiansen M, Tønder N, Larsen LA, Andersen PS, Simonsen H, Øyen N, et al. Mutations in the HERG K+-ion channel: A novel link between long QT syndrome and sudden infant death syndrome. Am J Cardiol. 2005;95:433–4. doi:10.1016/j.amjcard.2004.09.054.

111. Kong T, Feulefack J, Ruether K, Shen F, Zheng W, Chen X-Z, et al. Ethnic Differences in Genetic Ion Channelopathies Associated with Sudden Cardiac Death: A Systematic Review and Meta-Analysis. Ann Clin Lab Sci. 2017;47:481–90. http://www.ncbi.nlm.nih.gov/pubmed/28801377.

112. Iqbal Z, Vandeweyer G, van der Voet M, Waryah AM, Zahoor MY, Besseling JA, et al. Homozygous and heterozygous disruptions of ANK3: at the crossroads of neurodevelopmental and psychiatric disorders. Hum Mol Genet. 2013;22:1960–70. doi:10.1093/hmg/ddt043.

113. Kloth K, Denecke J, Hempel M, Johannsen J, Strom TM, Kubisch C, et al. First de novo ANK3 nonsense mutation in a boy with intellectual disability, speech impairment and autistic features. Eur J Med Genet. 2017;60:494–8. doi:10.1016/j.ejmg.2017.07.001.

114. Bi C, Wu J, Jiang T, Liu Q, Cai W, Yu P, et al. Mutations of ANK3 identified by exome sequencing are associated with autism susceptibility. Hum Mutat. 2012;33:1635–8. doi:10.1002/humu.22174.

115. Morgan AR, Hamilton G, Turic D, Jehu L, Harold D, Abraham R, et al. Association analysis of 528 intra-genic SNPs in a region of chromosome 10 linked to late onset Alzheimer’s disease. Am J Med Genet Part B Neuropsychiatr Genet. 2008;147B:727–31. doi:10.1002/ajmg.b.30670.

116. Wirgenes KV, Tesli M, Inderhaug E, Athanasiu L, Agartz I, Melle I, et al. ANK3 gene expression in bipolar disorder and schizophrenia. Br J Psychiatry. 2014;205:244–5. doi:10.1192/bjp.bp.114.145433.

117. Tesli M, Koefoed P, Athanasiu L, Mattingsdal M, Gustafsson O, Agartz I, et al. Association analysis of ANK3 gene variants in nordic bipolar disorder and schizophrenia case-control samples. Am J Med Genet Part B Neuropsychiatr Genet. 2011;156:969–74. doi:10.1002/ajmg.b.31244.

118. Yuan A, Yi Z, Wang Q, Sun J, Li Z, Du Y, et al. ANK3as a risk gene for schizophrenia: New data in han Chinese and meta analysis. Am J Med Genet Part B Neuropsychiatr Genet. 2012;159B:997–1005. doi:10.1002/ajmg.b.32112.

119. Orrù G, Carta MG. Genetic Variants Involved in Bipolar Disorder, a Rough Road Ahead. Clin Pract Epidemiol Ment Health. 2018;14:37–45. doi:10.2174/1745017901814010037.

120. Delvecchio G, Dima D, Frangou S. The effect of ANK3 bipolar-risk polymorphisms on the working memory circuitry differs between loci and according to risk-status for bipolar disorder. Am J Med Genet Part B Neuropsychiatr Genet. 2015;168:188–96. doi:10.1002/ajmg.b.32294.

121. Hori H, Yamamoto N, Teraishi T, Ota M, Fujii T, Sasayama D, et al. Cognitive effects of the ANK3 risk variants in patients with bipolar disorder and healthy individuals. J Affect Disord. 2014;158:90–6. doi:10.1016/j.jad.2014.02.008.

122. Lopez AY, Wang X, Xu M, Maheshwari A, Curry D, Lam S, et al. Ankyrin-G isoform imbalance and interneuronopathy link epilepsy and bipolar disorder. Mol Psychiatry. 2017;22:1464–72. doi:10.1038/mp.2016.233.

123. Ferreira MAR, O’Donovan MC, Meng YA, Jones IR, Ruderfer DM, Jones L, et al. Collaborative genome-wide association analysis supports a role for ANK3 and CACNA1C in bipolar disorder. Nat Genet. 2008;40:1056–8. doi:10.1038/ng.209.

124. Schulze TG, Detera-Wadleigh SD, Akula N, Gupta A, Kassem L, Steele J, et al. Two variants in Ankyrin 3 (ANK3) are independent genetic risk factors for bipolar disorder. Mol Psychiatry. 2009;14:487–91. doi:10.1038/mp.2008.134.

125. Zhu S, Cordner ZA, Xiong J, Chiu C-T, Artola A, Zuo Y, et al. Genetic disruption of ankyrin-G in adult mouse forebrain causes cortical synapse alteration and behavior reminiscent of bipolar disorder. Proc Natl Acad Sci. 2017;114:10479–84. doi:10.1073/pnas.1700689114.
