# Supplementary material for: An integrative methodology based on protein-protein interaction networks for identification and functional annotation of disease-relevant genes applied to channelopathies
Source: BMC Bioinformatics. 2019 Nov 12;20:565. doi: 10.1186/s12859-019-3162-1 (PMC6849233; doi:10.1186/s12859-019-3162-1)
Supplement: Supplementary file 8 — Additional file 8. Further functional annotation results after the exhaustive review of the most relevant genes in channelopathies. Summary of the localization, distribution and functions of the genes after the exhaustive review, as well as a summary of further information found in this review. CNS: Central Nervous System; PNS: Peripheral Nervous System; DRG: dorsal root ganglion. [file 12859_2019_3162_MOESM8_ESM.docx]

**Table 8.1.** **Summary of the localization, distribution and functions of the genes**. Table containing the functional annotation of the genes after the narrative review. CNS: Central Nervous System; PNS: Peripheral Nervous System; DRG: dorsal root ganglion.

| GENE | SUBUNIT ISOFORM | TISSUE AND SUBCELLULAR DISTRIBUTION | LOCATION | FUNCTION | REFERENCES |
| --- | --- | --- | --- | --- | --- |
| SCN1A | ALPHA NAV1.1 | CNS, PNS and cardiac myocytes. Soma and dendrites of expressing cells. | High expression:   - CNS, PNS - Cerebellum   Low expression:   - cardiac muscle - Striatum, hippocampus, thalamus | - Integration and propagation of synaptic inputs within the soma prior to the initiation and repetitive firing of axonal action potentials in neurons. - Excitation-contraction coupling in cardiac myocytes - Essential for normal response properties across species. | [1–7] |
| SCN2A | ALPHA NAV1.2 | CNS and PNS. Unmyelinated and premyelinated axons, axon initial segment (AIS) and terminals of expressing neurons. | High expression:   - central neurons | - Major component of the axonal transient, fast inactivating action potential conductance mechanism, as well as a regulator of neurotransmitter release in presynaptic terminals. - Action potential initiation and propagation, repetitive firing. | [1, 3, 5–9] |
| SCN4A/SCN4B | ALPHA NAV1.4/  NAVBETA4 | Skeletal muscle. | High expression:   - Adult skeletal muscle - Innervated adult myofibers   Low expression:   - Neonatal skeletal muscle   Absent in CNS, PNS and heart | - Generation and propagation of action potentials that initiate muscle contraction. | [5, 8, 10, 11] |
| SCN5A | ALPHA NA1.5 | Cardiac muscle, skeletal muscle and CNS.  Axons of mammalian brain. | High expression:   - Immature and denervated adult myofibers   Low expression:   - Brain (Adult DRG neurons, developing sensory neurons) - Skeletal muscle | - Mediates the fast inward sodium current (INa) that contributes to the rapid depolarization of the cardiac action potential. - Generates the conduction of cardiac impulse. - Initiation and propagation of action potentials. - Signals the initial heart beating, coordinating the contractions of upper and lower heart chambers and keeping a normal cardiac rhythm. | [1, 5, 8, 10, 12–18] |
| SCN9A | ALPHA NAV1.7 | PNS.  Somata of DRG neurons, along aβ-fibres and c fibres. | High expression:   - Nociceptive neurons: peripherally within free nerve endings in the epidermis; centrally within superficial lamina of the dorsal horn in the spinal cord - large and small diameter DRG neurons - aβ-fibres and c fibres - olfactory sensorial neurons   Absent in CNS | - Nociception signalling, regulating sensory neuron excitability and contributing to several sensory modalities. | [1, 5, 8, 19–22] |
| KCNQ2 | Kv7.2 | CN, PNS and others. Axonal Initial Segments (AISs), and nodes of Ranvier (NOR) (where Kv7.2/Kv7.3 channels are co-clustered with Nav channels) | Fetal, infant and adult:   - Neuronal tissues (such as sympathetic ganglia, hippocampus, cortex) - Lung - Testis   Fetal and adult: Heart, breast, eye, germ cell, placenta, small intestine | - Control of neuronal subthreshold excitability of neurons constructing a hetero-tetrameric channel with kv7.3 (encoded by KCNQ3) contributing to the naive M-current in brain - Maintenance of physiological functions of CNS controlling somatic excitability, bursting and neurotransmitter release - Foster the neuronal intrinsic firing activity in hippocampus and the slow after hyperpolarization which mediates the neuronal excitability | [23–27] |
| KCNH2 | Kv11.1 (hERG1) | Intracellular | - Cardiomyocytes (only cause disease in heart and epilepsy) - Nerve cells and microglia in CNS - Gastro-intestinal smooth muscle myocytes - Pancreatic beta-cells - Carotid body cells - Inner ear | - Forms the rapid component of the activating delayed-rectifier potassium current (IKr), which plays an essential role in the final repolarization of the ventricular action potential of vertebrate cardiomyocytes - Maintenance of the resting potential (QT interval) | [23, 27–30] |
| ANK3 | AnkG | Brain and neuromuscular junction.  Neuronal axon initial segments (AISs) and nodes of Ranvier (NOR) | General expression in neuronal and neuromuscular vertebral tissues | - Membrane-cytoskeleton adaptor that immobilizes integral membrane proteins to the spectrin-based membrane skeleton - Essential for neuronal axodendritic polarity, enabling action potential generations and conduction in neurons - Maintenance/targeting of cell adhesion molecules - Co-location of voltage-gated sodium channels (VGSCS) and KCNQ2/3 potassium channels to assemble and to maintain the neurotransmission at the distal AISs and nodes of Ranvier of neurons where the initiation of action potential happens and excitation/contraction coupling in muscle | [31–36] |

**8.2 Additional information**

These nine genes can be classified according to the genes families to which they belong. Thus, SCN1A, SCN2A, SCN4A, SCN4B, SCN5A and SCN9A genes belong to the voltage-gated sodium channels; KCNQ2 and KCNH2 genes are included in the voltage-gated potassium channels; and ANK3 gene belong to ankyrins family.

##### VOLTAGE-GATED SODIUM CHANNELS

Voltage-gated sodium (*Nav*) channels (VGSC) are heteromultimeric membrane proteins. These proteins comprise a large alpha subunit, forming the pore with voltage-dependent gating and selectivity for sodium, and one or more accessory beta subunits, controlling the expression and activity of the pore-forming subunit [5, 37]. Ten genes encode an alpha unit in mammals, nine of them closely related [5]. Sodium currents are encoded by at least four α subunits (SCN1A, SCN2A, SCN3A and SCN8A) [37]. Voltage-gated sodium channels mediate the selective pass of sodium ions in accordance with their electrochemical gradient controlling the sodium flux between cells, assuming opened or closed conformations in response to the voltage difference across the cellular membrane [12].

Voltage-gated sodium-selective ion channels are present in the membrane of most excitable cells (in both neurons and glia [5, 6], including central and peripheral nervous system (SCN1A, SCN2A, SCN3A, SCN6A; only peripheral: SCN7A, SCN8A, SCN9A [5]), muscle (SCN4A, SCN5A [5]) and neuroendocrine cell types. They are low expressed in non-excitable cells with an unclear role [7].

These channels play a key role in the generation and propagation of neural action potential and communication, cardiac excitation-contraction coupling, and skeletal and intestinal function [38]. In the heart, *Nav* channels produce the rapid upstroke of cardiac action potential that is essential for the rhythmic beating of the heart [18, 39]. In the innervated and denervated skeletal muscle, these channels achieve the initiation and propagation of action potential, allowing the activation of somatosensory neurons which induce pain without neurogenic inflammation and result in hypersensitivity to mechanical stimuli, but not thermal [40]. In the brain these channels are the major mediators of normal neuronal firing [37] determining the release of neurotransmitters [40].

##### VOLTAGE-GATED POTASSIUM CHANNELS

The most heterogeneous class of ion channels is the voltage-gated potassium (*Kv*) channels (VGKC) [27], which regulate the excitability of nervous system by controlling the action potential duration, the subthreshold electrical properties and the responsiveness to synaptic inputs [41]. The transport outside neurons of potassium ions positively charged by heterotetramerization of KCNQ2/KCNQ3 (functional KCNQ channels) give rise to the transmission of a slowly activating and deactivating potassium conductance called M-current (IM), described in CNS and PNS. This muscarinic-regulated K+ current is one of the most important regulator of the subthreshold neuronal excitability in many human neurons [26, 42]. This current also determines the responsiveness to synaptic inputs [27] repolarizing the neuronal membrane back toward resting membrane potential during long-lasting depolarizing inputs, limiting repetitive firing and causing spike-frequency adaptation [41]. Finally, M-current prevents and controls seizure activity possibly being involved in learning, memory [25, 27] and epilepsy [26].

KCNQ2 encodes for the delayed rectifier protein Kv7.2 [27], a slow activating and non-activating voltage-gated potassium channel expressed in several neuronal populations. KCNQ2 protein is expressed in some presynaptic terminals and it is also co-localized with KCNQ3 protein in the soma and dendrites on pyramidal and polymorphic neurons in the cortex and hippocampus [27]. KCNQ2 mutations influence on the M current leading to neurological diseases such as epilepsy [24], so that M current is considered a new target for antiepileptic therapy [27]. In fact, KCNQ2 and KCNQ3 have the closest correlation with epilepsy in the KCNQ family.

KCNH2 encodes for the rapid component of the delayed rectifier potassium current (IKr) ion channel Kv11.1 (hERG1) [27, 30], which mediates the action potential repolarization in the heart [30]. Co-assembly of KCNH2 with the MiRP1 subunit (KCNE2) is required to fully reproduce the biophysical and pharmacological properties of the native IKr [43]. Fast onset of inactivation of KCNH2 channels causes rectification of IKr at depolarized potentials. IKr current also displays a rapid recovery from inactivation followed by slower deactivation kinetics upon repolarization. Because of these unique properties, the contribution of IKr to the early plateau phases (1 and 2) of the ventricular action potential remains minimal but gradually increases with repolarization to peak near the transition between phase 2 and phase 3 of the action potential [43].

KCNH2 mutations are associated with different levels of risk for cardiac arrhythmias (as ventricular arrhythmia and sudden cardiac death) in long QT syndrome, which causes a disorder of cardiac repolarization. KCNH2 mutations also predisposes affected individuals to epilepsy and short QT syndrome [30, 44].

##### ANKYRIN G PROTEINS

Ankyrins (*ANKG*) are a family of multifunctional membrane adaptor proteins ubiquitously expressed throughout the brain and the neuromuscular junction [31, 35]. Within this family there is a class of membrane-cytoskeletal scaffold protein called Ankyrin G (AnkG) encoded by Ank3 gene [31–34].

Ankyrin G proteins binds vertebrate KCNQ channels (either KCNQ2/KCNQ3 heteromers, or KCNQ2 homomers) and Na_v_ channel α-subunits (Nav 1.1, Nav 1.2 and Nav1.6, depending on cell type), all concentrated at AISs and nodes of Ranvier, playing a role in the axodendritic polarity mediating concentration of those channels. It allows the generation and conduction of action potentials through myelinated axons in many mammalian neurons [35, 36]. AnkG proteins also interact with a variety of other integral membrane proteins (CAMs neurofascin/ NrCAM) [35].

**References**

1. Trimmer JS, Rhodes KJ. Localization of voltage-gated ion channels in mammalian brain. Annu Rev Physiol. 2004;66:477–519. doi:10.1146/annurev.physiol.66.032102.113328.

2. Yates A, Akanni W, Amode MR, Barrell D, Billis K, Carvalho-Silva D, et al. Ensembl 2016. Nucleic Acids Res. 2016;44:D710–6. doi:10.1093/nar/gkv1157.

3. Leary RJ, Kinde I, Diehl F, Schmidt K, Clouser C, Duncan C, et al. Development of personalized tumor biomarkers using massively parallel sequencing. Sci Transl Med. 2010;2:20ra14. doi:10.1126/scitranslmed.3000702.

4. Meisler MH, Kearney JA. Sodium channel mutations in epilepsy and other neurological disorders. J Clin Invest. 2005;115:2010–7. doi:10.1172/JCI25466.

5. Schaller KL, Caldwell JH. Expression and distribution of voltage-gated sodium channels in the cerebellum. Cerebellum. 2003;2:2–9. doi:10.1080/14734220309424.

6. Plummer NW, Meisler MH. Evolution and Diversity of Mammalian Sodium Channel Genes. Genomics. 1999;57:323–31. doi:10.1006/geno.1998.5735.

7. Catterall WA. International Union of Pharmacology. XLVII. Nomenclature and Structure-Function Relationships of Voltage-Gated Sodium Channels. Pharmacol Rev. 2005;57:397–409. doi:10.1124/pr.57.4.4.

8. Meisler MH, O’Brien JE, Sharkey LM. Sodium channel gene family: epilepsy mutations, gene interactions and modifier effects. J Physiol. 2010;588:1841–8. doi:10.1113/jphysiol.2010.188482.

9. Oliva M, Berkovic SF, Petrou S. Sodium channels and the neurobiology of epilepsy. Epilepsia. 2012;53:1849–59. doi:10.1111/j.1528-1167.2012.03631.x.

10. Loussouarn G, Sternberg D, Nicole S, Marionneau C, Le Bouffant F, Toumaniantz G, et al. Physiological and Pathophysiological Insights of Nav1.4 and Nav1.5 Comparison. Front Pharmacol. 2016;6 JAN. doi:10.3389/fphar.2015.00314.

11. Corrochano S, Männikkö R, Joyce PI, McGoldrick P, Wettstein J, Lassi G, et al. Novel mutations in human and mouse SCN4A implicate AMPK in myotonia and periodic paralysis. Brain. 2014;137:3171–85. doi:10.1093/brain/awu292.

12. Chagot B, Potet F, Balser JR, Chazin WJ. Solution NMR Structure of the C-terminal EF-hand Domain of Human Cardiac Sodium Channel Na V 1.5. J Biol Chem. 2009;284:6436–45. doi:10.1074/jbc.M807747200.

13. Wu L, Nishiyama K, Hollyfield JG, Wang Q. Localization of Nav1.5 sodium channel protein in the mouse brain. Neuroreport. 2002;13:2547–51. doi:10.1097/01.wnr.0000052322.62862.a5.

14. Kinoshita K, Takahashi H, Hata Y, Nishide K, Kato M, Fujita H, et al. SCN5A(K817E), a novel Brugada syndrome–associated mutation that alters the activation gating of NaV1.5 channel. Hear Rhythm. 2016;13:1113–20. doi:10.1016/j.hrthm.2016.01.008.

15. Gellens ME, George AL, Chen LQ, Chahine M, Horn R, Barchi RL, et al. Primary structure and functional expression of the human cardiac tetrodotoxin-insensitive voltage-dependent sodium channel. Proc Natl Acad Sci U S A. 1992;89:554–8. doi:10.1073/pnas.89.2.554.

16. Hu W, Tian C, Li T, Yang M, Hou H, Shu Y. Distinct contributions of Na(v)1.6 and Na(v)1.2 in action potential initiation and backpropagation. Nat Neurosci. 2009;12:996–1002. doi:10.1038/nn.2359.

17. Domínguez JN, de la Rosa Á, Navarro F, Franco D, Aránega AE. Tissue distribution and subcellular localization of the cardiac sodium channel during mouse heart development. Cardiovasc Res. 2008;78:45–52. doi:10.1093/cvr/cvm118.

18. Wang L, Meng X, Yuchi Z, Zhao Z, Xu D, Fedida D, et al. De Novo Mutation in the SCN5A Gene Associated with Brugada Syndrome. Cell Physiol Biochem. 2015;36:2250–62. doi:10.1159/000430189.

19. Stamboulian S, Choi J-S, Ahn H-S, Chang Y-W, Tyrrell L, Black JA, et al. ERK1/2 Mitogen-Activated Protein Kinase Phosphorylates Sodium Channel Nav1.7 and Alters Its Gating Properties. J Neurosci. 2010;30:1637–47. doi:10.1523/JNEUROSCI.4872-09.2010.

20. Dib-Hajj SD, Yang Y, Black JA, Waxman SG. The NaV1.7 sodium channel: from molecule to man. Nat Rev Neurosci. 2012;14:49–62. doi:10.1038/nrn3404.

21. Ahn H-S, Black J a, Zhao P, Tyrrell L, Waxman SG, Dib-Hajj SD. Nav1.7 is the predominant sodium channel in rodent olfactory sensory neurons. Mol Pain. 2011;7:32. doi:10.1186/1744-8069-7-32.

22. Meguro K, Iida H, Takano H, Morita T, Sata M, Nagai R, et al. Function and role of voltage-gated sodium channel Na V 1.7 expressed in aortic smooth muscle cells. Am J Physiol Circ Physiol. 2009;296:H211–9. doi:10.1152/ajpheart.00960.2008.

23. Trimmer JS, Messing A, Chiu SY, Jan LY, Sakaguchi I, Katsuura G, et al. Subcellular Localization of K+ Channels in Mammalian Brain Neurons: Remarkable Precision in the Midst of Extraordinary Complexity. Neuron. 2015;85:238–56. doi:10.1016/j.neuron.2014.12.042.

24. Abidi A, Devaux JJ, Molinari F, Alcaraz G, Michon F-X, Sutera-Sardo J, et al. A recurrent KCNQ2 pore mutation causing early onset epileptic encephalopathy has a moderate effect on M current but alters subcellular localization of Kv7 channels. Neurobiol Dis. 2015;80:80–92. doi:10.1016/j.nbd.2015.04.017.

25. Steinlein OK, Stoodt J, Biervert C, Janz D, Sander T. The voltage gated potassium channel KCNQ2 and idiopathic generalized epilepsy. Neuroreport. 1999;10:1163–6. http://www.ncbi.nlm.nih.gov/pubmed/10363917.

26. Diao Y, Tian Y, Han S, Zhang N, Li J, Yin Y. Current insight into the role of voltage-gated potassiumion channel 7 (Kv7) channels: an emerging therapy target against epilepsy. Neuropsychiatry (London). 2017;07:26–31. doi:10.4172/Neuropsychiatry.1000175.

27. Gutman GA. International Union of Pharmacology. LIII. Nomenclature and Molecular Relationships of Voltage-Gated Potassium Channels. Pharmacol Rev. 2005;57:473–508. doi:10.1124/pr.57.4.10.

28. Gianulis EC, Trudeau MC. Rescue of Aberrant Gating by a Genetically Encoded PAS (Per-Arnt-Sim) Domain in Several Long QT Syndrome Mutant Human Ether-á-go-go -related Gene Potassium Channels. J Biol Chem. 2011;286:22160–9. doi:10.1074/jbc.M110.205948.

29. Ng CA, Phan K, Hill AP, Vandenberg JI, Perry MD. Multiple interactions between cytoplasmic domains regulate slow deactivation of Kv11.1 channels. J Biol Chem. 2014;289:25822–32. doi:10.1074/jbc.M114.558379.

30. Sanguinetti MC. HERG1 channelopathies. Pflugers Arch. 2010;460:265–76. doi:10.1007/s00424-009-0758-8.

31. Liu Y, Zhang Y, Wang J-H. Crystal structure of human Ankyrin G death domain. Proteins. 2014;82:3476–82. doi:10.1002/prot.24702.

32. Hori H, Yamamoto N, Teraishi T, Ota M, Fujii T, Sasayama D, et al. Cognitive effects of the ANK3 risk variants in patients with bipolar disorder and healthy individuals. J Affect Disord. 2014;158:90–6. doi:10.1016/j.jad.2014.02.008.

33. Wirgenes KV, Tesli M, Inderhaug E, Athanasiu L, Agartz I, Melle I, et al. ANK3 gene expression in bipolar disorder and schizophrenia. Br J Psychiatry. 2014;205:244–5. doi:10.1192/bjp.bp.114.145433.

34. Lopez AY, Wang X, Xu M, Maheshwari A, Curry D, Lam S, et al. Ankyrin-G isoform imbalance and interneuronopathy link epilepsy and bipolar disorder. Mol Psychiatry. 2017;22:1464–72. doi:10.1038/mp.2016.233.

35. Lemaillet G, Walker B, Lambert S. Identification of a conserved ankyrin-binding motif in the family of sodium channel alpha subunits. J Biol Chem. 2003;278:27333–9. doi:10.1074/jbc.M303327200.

36. Xu M, Cooper EC. An Ankyrin-G N-terminal Gate and Protein Kinase CK2 Dually Regulate Binding of Voltage-gated Sodium and KCNQ2/3 Potassium Channels. J Biol Chem. 2015;290:16619–32. doi:10.1074/jbc.M115.638932.

37. Shi X, Yasumoto S, Kurahashi H, Nakagawa E, Fukasawa T, Uchiya S, et al. Clinical spectrum of SCN2A mutations. Brain Dev. 2012;34:541–5. doi:10.1016/j.braindev.2011.09.016.

38. Musa H, Kline CF, Sturm AC, Murphy N, Adelman S, Wang C, et al. SCN5A variant that blocks fibroblast growth factor homologous factor regulation causes human arrhythmia. Proc Natl Acad Sci U S A. 2015;112:12528–33. doi:10.1073/pnas.1516430112.

39. Zeng Z, Zhou J, Hou Y, Liang X, Zhang Z, Xu X, et al. Electrophysiological Characteristics of a SCN5A Voltage Sensors Mutation R1629Q Associated With Brugada Syndrome. PLoS One. 2013;8:e78382. doi:10.1371/journal.pone.0078382.

40. UniProtKB. http://www.uniprot.org/uniprot/.

41. Castaldo P, del Giudice EM, Coppola G, Pascotto A, Annunziato L, Taglialatela M. Benign familial neonatal convulsions caused by altered gating of KCNQ2/KCNQ3 potassium channels. J Neurosci. 2002;22:RC199. doi:20025989.

42. Milh M, Lacoste C, Cacciagli P, Abidi A, Sutera-Sardo J, Tzelepis I, et al. Variable clinical expression in patients with mosaicism for KCNQ2 mutations. Am J Med Genet Part A. 2015;167:2314–8. doi:10.1002/ajmg.a.37152.

43. Cordeiro JM, Brugada R, Wu YS, Hong K, Dumaine R, Gussak I, et al. Modulation of I(Kr) inactivation by mutation N588K in KCNH2: a link to arrhythmogenesis in short QT syndrome. Cardiovasc Res. 2005;67:498–509. doi:10.1016/j.cardiores.2005.02.018.

44. Moss AJ, Kass RS. Long QT syndrome: from channels to cardiac arrhythmias. J Clin Invest. 2005;115:2018–24. doi:10.1172/JCI25537.
